# Revisiting the relationships among howler monkeys through molecular phylogenetic analysis (Primates; Atelidae; *Alouatta*)

**DOI:** 10.1007/s10329-025-01233-0

**Published:** 2026-01-20

**Authors:** Danillo Figueiredo da Silva, Rodrigo Petry Corrêa de Sousa, Adam Bessa-Silva, Grazielle Fernanda Evangelista Gomes, Marcelo Vallinoto, Iracilda Sampaio

**Affiliations:** 1 Laboratório de Evolução, Instituto de Estudos Costeiros, Universidade Federal do Pará, Campus de Bragança, Bragança, PA Brazil; 2 Laboratório de Genética Aplicada, Instituto de Estudos Costeiros, Universidade Federal do Pará, Campus de Bragança, Bragança, PA Brazil

**Keywords:** Amazon, Multilocus, Neotropical primates, Platyrrhini, Pliocene

## Abstract

The howler monkeys (*Alouatta*) are part of the Platyrrhini infraorder, a group of Neotropical primates with the widest geographical distribution, occurring from southern Mexico to northern Argentina. Previous phylogenetic studies based on cytogenetic, morphological, and molecular data have shown inconsistent results, requiring new approaches to clarify relationships within the genus. In this study, we analyzed nine species of *Alouatta* using 24 molecular markers (2 mitochondrial and 22 nuclear genes). Through Bayesian inference, Maximum Likelihood, and divergence time analyses, we inferred the phylogeny and estimated the timing of speciation events. Our results recovered two major clades within *Alouatta*, corresponding to Mesoamerican (Trans-Andean) and South American (Cis-Andean) lineages. Most diversification events occurred during the Pliocene. Within the South American clade, we identified two well-supported groups: one composed of species from the Atlantic Forest and eastern Amazon (*A. guariba*, *A. belzebul*, and *A. discolor*), likely shaped by the formation of the South American dry diagonal; and another formed by *A. caraya* and the *A. seniculus* complex. Notably, our results confirmed a close phylogenetic relationship between *A. discolor* and *A. belzebul*, and between *A. macconnelli* and *A. nigerrima*, which occur on opposite sides of the Amazon River.These relationships, along with the confirmation of *A. discolor* and *A. nigerrima* as distinct species based on multilocus evidence from known localities, represent advances over previous studies and contribute to a more resolved understanding of *Alouatta* diversification.

**Supplementary Information:**

The online version contains supplementary material available at 10.1007/s10329-025-01233-0.

## Introduction

**Fig. 1:**
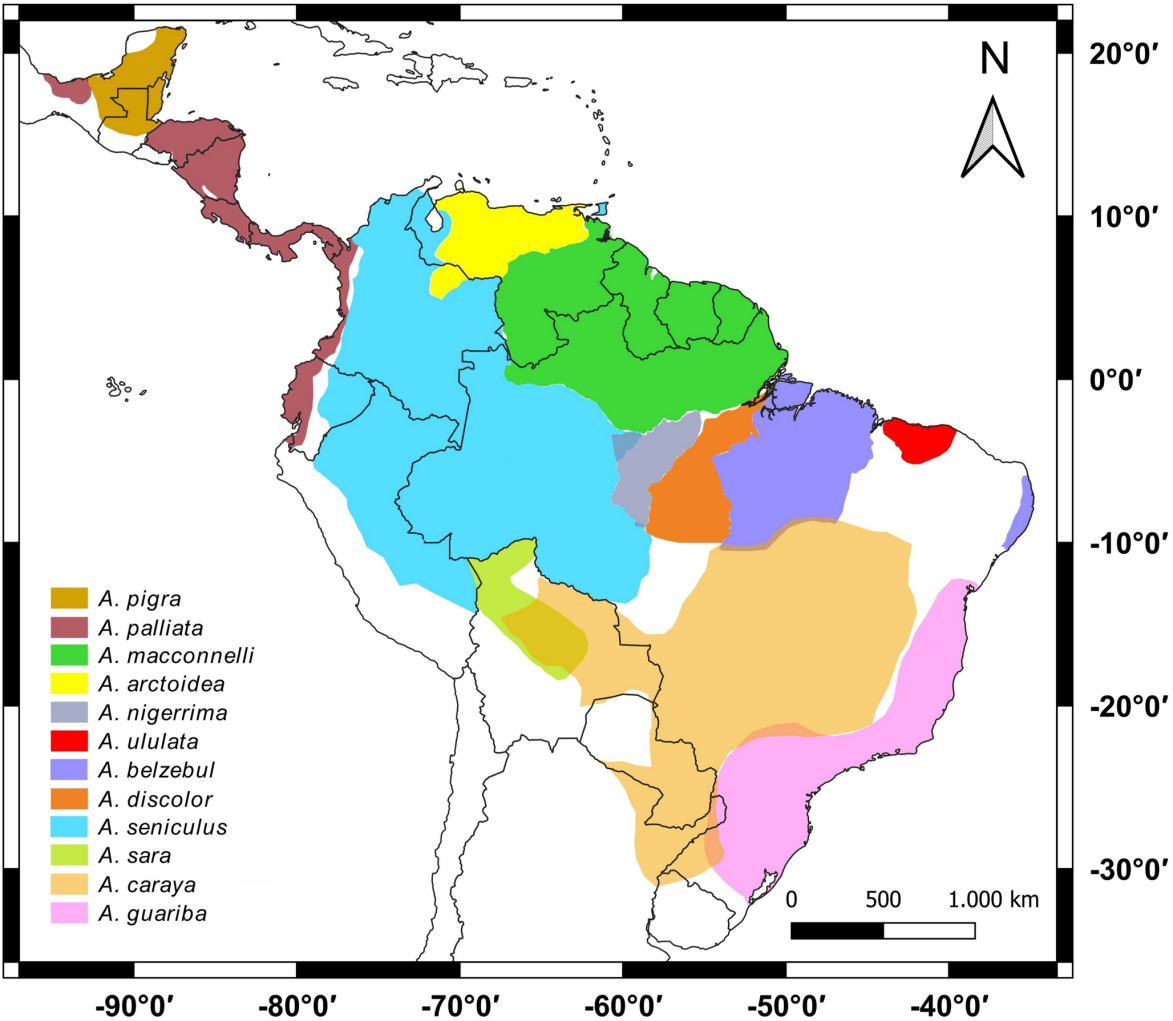
Geographical distribution of the recognized species of the genus, according to Cortés-Ortiz et al. (2015). Updated geographical information is adapted from the IUCN Red List of Threatened Species (2025)

Howler monkeys are Neotropical primates of the genus *Alouatta*, which have the largest geographical distribution within the Platyrrhini, occurring from Vera Cruz, Mexico to Corrientes, Argentina (Cabrera 1939; Estrada and Coates-Estrada 1984) (Fig. 1). These Neotropical primates are the largest monkeys in the New World, along with the other genera of Atelidae. These monkeys, which eat leaves and fruits, are ecologically important for floristic succession and maintaining the biodiversity of ecosystems (Bravo 2022). They are animals that inhabit different types of ecosystems and habitats, from tropical forests, such as the Amazon and the Atlantic Rainforest, to open areas, such as the Caatinga and the Cerrado (Gregorin 2006). However, these primates have suffered constant population declines and even local extinctions due to extreme habitat loss and alteration, leading many *Alouatta* species to the status of vulnerable or endangered to extinction (IUCN 2025).

The taxonomic history of the genus *Alouatta* is long and complex, involving a lot of problematic data on the evolutionary relationships of these primates (Cortés-Ortiz et al. 2015a, b; Schwantes et al. 2025). Although different studies have been carried out based on morphological, cytogenetic, and molecular data to infer the relationships among howler monkeys, the divergent results still generate enormous confusion about the validity of many taxa within the genus. Hershkovitz (1949), in his systematic review of the genus *Alouatta*, initially separated the genus into three groups: the *Alouatta seniculus* group (*A. seniculus*,* Alouatta belzebul*, and *Alouatta fusca*); the *Alouatta palliata* group (*A. palliata* and *Alouatta pigra*), and the *Alouatta caraya* group (*A. caraya*). These groups were also recognized by other authors later (Hill 1962; Rylands and Mittermeier 2009). However, numerous reclassifications occur among the groups and species of *Alouatta*, and currently, the most recent study of the taxonomy review considers 16 taxa for the genus, and 11 of them are monotypic species (Rylands and Mittermeier 2024).

The monophyly of *Alouatta* has been recognized by different studies (Cortés-Ortiz et al. 2003; Doyle et al. 2021; Perelman et al. 2011; Schwantes et al. 2025). Although little consensus has been reached on the phylogenetic relationships within *Alouatta*, the most recent studies based on a large dataset have recovered the division of the genus into two monophyletic groups, the South American or Cis-Andean clade and the Mesoamerican or Trans-Andean clade (Cortés-Ortiz et al. 2003; Doyle et al. 2021; Kuderna et al. 2023; Schwantes et al. 2025).

The main problems observed in the relationships among *Alouatta* species can be due to the use of a limited number of markers, limited taxonomic sampling, different species used, and/or the different methods employed (cytogenetics, molecular, and morphology) (Bonvicino et al. 2001; Cortés-Ortiz et al. 2003; Doyle et al. 2021; Gregorin 2006; Kuderna et al. 2023; Meireles et al. 1999; Oliveira et al. 2002; Schwantes et al. 2025).

The study of Cortés-Ortiz et al. (2003) included species from Central America and part of the taxa from the South American region, and which separated the genus for the first time into a Mesoamerican clade and a well-supported South American clade, where two monophyletic groups within the South American clade were also recovered. However, although it was a multilocus study with mitochondrial and nuclear data, the number of markers used was considerably lower compared to more recent research.

More recent studies, such as Doyle et al. (2021) and Kuderna et al. (2023), although they corroborate the separation of *Alouatta* into two groups (Mesoamerican and South American clades), based their molecular analyses solely on nuclear data from intergenic regions and ultraconserved elements of the genome, respectively.

In contrast, Povill et al. (2022, 2023**)** analyzed partial mitochondrial sequences, mainly of the *cytochrome b* gene, to infer phylogenetic relationships within the genus. Despite recovering the major Mesoamerican and South American divisions, the reduced dataset and limited taxonomic sampling resulted in topologies that differ from those obtained in broader multilocus analyses. These discrepancies likely reflect the influence of single-locus phylogenetic noise and the absence of representative samples from key geographic regions, particularly in South America.

Additionally, although Schwantes et al. (2025) employed both nuclear and mitochondrial markers, their dataset consisted exclusively of sequences available in GenBank, most of which lacked precise geographic and voucher information. This restricted sampling design prevents the verification of specimen identity, hinders the association between genetic lineages and their geographic ranges, and may therefore reduce the reliability of their taxonomic and biogeographic inferences. Furthermore, in these studies, the relationships within *Alouatta* were not congruent with each other, especially in relation to the South American clade. In turn, in the study by Janiak et al. (2022) and Povill et al. (2025), using mitochondrial genomes, the phylogeny recovered for *Alouatta* was similar to the phylogenetic arrangement obtained by Cortés-Ortiz et al. (2003), where the main differences were the result of the different numbers of taxa used in each study.

Based on the observation of the data described above, it is clear that there are numerous incongruence between the studies regarding the phylogenetic relationships of howler monkeys. Furthermore, the problems are not limited to phylogenetic relationships; different dates for the separation of the Mesoamerican and South American clades of *Alouatta* are observed in different studies, and there is no consensus, which limits a better understanding of the divergence of *Alouatta* species (Cortés-Ortiz et al. 2003; Doyle et al. 2021; Kuderna et al. 2023).

These inconsistencies in estimates of divergence time between previous studies likely stem from differences in the number and type of molecular markers analyzed, the calibration strategies employed, and the use of different molecular clock models. For example, some analyses were based exclusively on mitochondrial genomes or ultra-conserved elements, while others used a single fossil calibration point or inconsistent calibrations, which can produce time estimates that are not directly comparable (Cortés-Ortiz et al. 2003; Doyle et al. 2021; Janiak et al. 2022; Kuderna et al. 2023; Povill et al. 2025; Schwantes et al. 2025).

Thus, considering these limitations, our study presents an evaluation of the evolutionary relationships within the genus *Alouatta* based on a combined analysis of nuclear and mitochondrial markers from verified specimens, applying four independent fossil calibrations within a relaxed clock framework. By integrating multiple data sources and analytical approaches, our research provides a robust and consistent temporal framework to clarify systematic uncertainties and to better understand the natural history and evolutionary processes that shaped the diversification of these Neotropical primates.

## Materials and methods

### Biological sampling, extraction, amplification, and DNA sequencing

Blood and/or muscle tissue samples were obtained from 37 individuals of a total of 6 *Alouatta* species (*A. belzebul*,* A. caraya*,* Alouatta discolor*,* Alouatta guariba*, *Alouatta macconnelli*, and *Alouatta nigerrima*) and two individuals, one belonging to *Ateles paniscus* and the other to *Lagothrix lagotricha* (outgroup), from the South American region. These samples were obtained from the DNA databanks of the Universidade Federal do Pará-Campus Bragança, and they have already been referenced in previous studies (Rocha et al. 1990; Sampaio et al. 1991; Schneider et al. 1994). For the species *A. palliata*,* A. pigra*, and *A. sara*, we used DNA sequences retrieved from the GenBank database, as well as for the species that comprise the outgroup, which corresponds to two species of the Atelidae family (*Ateles geoffroyi* and *Brachyteles arachnoides*) (Table S1). Our dataset initially included two sequences attributed to *A. sara* (Asar and Asa). The Asa record, however, comes from GenBank without any information on its geographic origin. To avoid discarding it prematurely, we kept Asa only in the BI and ML phylogenetic analyses to check whether it grouped with the verified sample (Asar). Although it consistently clustered with Asar, the lack of provenance made it unreliable for downstream analyses. For this reason, Asa was excluded from all subsequent steps to minimize potential bias and maintain the reliability of the dataset. It should be noted that two sequences attributed to *A. sara* (Asar and Asa) were initially included in the dataset. However, the Asa sequence corresponds to a GenBank record without geographic information. For this reason, Asa was retained only in the Baysean Inference (BI) and Maximum likelihood (ML) phylogenetic analyses to confirm its position in relation to the verified sample (Asar). As it consistently clustered with Asar but remained unverifiable, Asa was excluded from all subsequent analyses to avoid possible biases and ensure data reliability.

Total DNA from blood and muscle tissue samples was extracted using the Wizard Genomic Kit (Promega Corporation, Madison, WI, USA) according to the manufacturer’s guidelines. The DNA molecular markers used in this study corresponded to 22 nuclear and 2 mitochondrial genes, chosen from the studies by Kiesling et al. (2015) and Perelman et al. (2011) (Table S3). These markers were amplified via polymerase chain reaction (PCR). Each reaction was performed in a volume of 15 µL containing 1 µL of genomic DNA; 2.4 µL of dNTPs (1.25 mM); 1.5 µL of 10x buffer; 0.6 µL of MgCl_2_ (25 mM); 0.6 µL of each primer (50 ng/µL); 0.8 µL of DNA and 0.1 µL of Taq DNA Polymerase. The PCR protocol was the following: 3 min of initial denaturation, followed by 35 steps, each with 30 s of denaturation at 94 °C, 45 s of hybridization temperature at a variable temperature depending on the primer (Table S3), and 1 min of extension at 72 °C. Finally, we used a final extension of 4 min at 72 °C, after the previous 35 steps.

PCR positives were purified using the protocol with PEG (polyethylene glycol) and ethyl alcohol (Paithankar and Prasad 1991). The sequencing of these molecular markers was implemented using the sequencing method of Sanger et al. (1977) on an ABI 3500 automatic sequencer (Applied Biosystems, Foster City, CA, USA). All the sequenced samples have been deposited in GenBank (Table S2) and data for species, their codes, accession numbers, and geographical origins can be seen in Table 1.

**Table 1:** Species sequenced in this study, identification codes, and geographical origin

Species	Vouchers	Geographical origin
*A. belzebul*	AbBrag	Bragança–Pará, Brasil
*A. belzebul*	AbCAX	Floresta Caxiuanã–Pará, Brasil
*A. belzebul*	AbANP	Anapu–Pará, Brasil
*A. belzebul*	Ab297, Ab516, Ab1252, Ab1509	Tucuruí–Pará, Brasil
*A. belzebul*	Ab38	Xingu, Leste–Pará, Brasil
*A. macconnelli*	Am2230, Am2502, Am2532, Am2538	Balbina, Leste–Amazonas, Brasil
*A. macconnelli*	Am2524,	Balbina, Direita–Amazonas, Brasil
*A. macconnelli*	Am2090	Jari, Leste–Amapá, Brasil
*A. macconnelli*	Am2094, Am2096, Am2100	Jari, Oeste–Pará, Brasil
*A. macconnelli*	Am3087	Cachoeira Porteira–Pará, Brasil
*A. discolor*	Ad	Marajó–Pará, Brasil
*A. nigerrima*	An65, An84	Itaituba–Pará, Brasil
*A. nigerrima*	An10	Unknown
*A. caraya*	Aca01, Aca02, Aca03, Aca04	Serra da Mesa–Goiás, Brasil
*A. caraya*	Aca38, Aca55, Aca58, Aca68, Aca73, Aca80	Centro Argentino de Primatas (CAPRIM)
*A. guariba*	Ag35, Ag43, Ag44, Ag45, Ag46	Santa Catarina, Brasil
*A. paniscus*	Atpan	Unknown
*L. lagotricha*	Lla	Unknown

### Sequence alignment and evolutionary models

The sequences were aligned in ClustalW (Thompson et al. 1994), after visual inspection of the quality of the electropherograms of the molecular marker fragments in the BioEdit software (Hall 1999). Phylogenetic analyses were conducted using a partitioned dataset, in which each gene fragment was treated as an independent partition with its respective evolutionary model, as indicated in Table S4. Model selection followed the Bayesian Information Criterion (BIC) implemented in Kakusan4 program (Tanabe 2011). The levels of variation for each marker were described using the MEGA11 software (Tamura et al. 2021), where the number of variable and informative sites for parsimony were calculated (Table S4). In addition, we calculate by MEGA11 software, the mean genetic distances among *Alouatta* species, using the K2P model, with concatenated nuclear + mitochondrial DNA data (Table 2), mitochondrial data (Table S5), and nuclear data (Table S6).

**Table 2:** Mean genetic distance among *Alouatta* species and other genera of the Atelidae family (*Ateles paniscus*, *Brachyteles arachnoides*, and *Lagothrix lagotricha*)

	1	2	3	4	5	6	7	8	9	10	11
1 - *Alouatta belzebul*											
2 - *Alouatta discolor*	0.0028										
3 - *Alouatta guariba*	0.0113	0.0157									
4 - *Alouatta nigerrima*	0.0112	0.0168	0.0116								
5 - *Alouatta macconnelli*	0.0115	0.0170	0.0121	0.0014							
6 - *Alouatta caraya*	0.0122	0.0176	0.0131	0.0106	0.0111						
7 - *Alouatta palliata*	0.0171	0.0234	0.0172	0.0170	0.0171	0.0180					
8 - *Alouatta sara*	0.0130	0.0271	0.0163	0.0089	0.0086	0.0123	0.0226				
9 - *Alouatta pigra*	0.02	0.0294	0.0221	0.0240	0.0234	0.0238	0.0156	0.0481			
10 - *Lagothrix lagotricha*	0.0436	0.0536	0.0460	0.0443	0.0452	0.0457	0.0473	0.0632	0.0683		
11 - *Ateles paniscus*	0.0432	0.0556	0.0442	0.0434	0.0437	0.0437	0.0463	0.0501	0.0521	0.0351	
12 - *Brachyteles arachnoides*	0.0473	0.0603	0.0477	0.0458	0.0472	0.0472	0.0508	0.0514	0.0592	0.0352	0.0362

Based on concatenated mitochondrial and nuclear data, using the K2P model

### Phylogenetic analysis and estimation of divergence times

Phylogenetic analyses were performed using the concatenated sequence matrix, as well as separate datasets comprising only mitochondrial DNA and only nuclear DNA markers. Phylogenetic relationships were estimated using the Maximum likelihood and Bayesian inference methods. The Maximum likelihood analyses were carried out using the RAxML v.8.2.12 software (Stamatakis 2014) with 1000 bootstrap pseudoreplicates. In turn, Bayesian inference was performed in the program MrBayes v.3.2.7 (Ronquist and Huelsenbeck 2003) with two independent Markov Chains Monte Carlo (MCMC). A total of 10 million generations were used, with sampling every 1000 and a burn-in of 20%. The analyses for both RAxML and MrBayes were performed on the CIPRES platform (Miller et al. 2010).

Phylogenetic inference analyses and divergence time estimation were also done using the BEAST v.1.8.4 software (Drummond et al. 2012). We used a relaxed uncorrelated molecular clock with 20 million generations of MCMC to estimate the phylogenetic tree. The quality of the run was evaluated in Tracer v.1.7 (Rambaut et al. 2018), where we measured ESS values > 200, indicating convergence of the chain. The tree file was summarized using TreeAnnotator v.1.8 (Drummond et al. 2012).

For the divergence time analysis, we used a relaxed uncorrelated molecular clock run using the log-normal model. The Yule speciation process was used as the prior of the tree. We used four calibration points to calculate the divergence times: (1) the separation between *Ateles paniscus* and *Ateles geoffroyi* estimated at around 3.7 million years (Kiesling et al. 2015; Morales-Jimenez et al. 2015); (2) the separation between *Ateles* and *Lagothrix* + *Brachyteles* estimated at 10.7 million years (Kiesling et al. 2015); (3) the separation between *Lagothrix* and *Brachyteles* estimated at around 9.5 million years (Kiesling et al. 2015; Perelman et al. 2011), and (4) the fossil *Stirtonia tatacoensis*, dating back 12.6–13.7 million years (Rosenberger et al. 2015), as a calibration point for the node grouping *Alouatta*, and its sister group (*Lagothrix*,* Brachyteles*, and *Ateles*). The fossil *Stirtonia tatacoensis* (Kay et al. 1987) from the La Venta Formation was used to calibrate the crown node of Atelidae (including *Alouatta*, *Lagothrix*, *Brachyteles*, and *Ateles*), following previous studies (Kiesling et al. 2015; Morales-Jiménez et al. 2015; Rosenberger et al. 2015). Although the precise phylogenetic position of *Stirtonia* within Alouattinae remains debated, its morphological affinities with *Alouatta* justify its use as a conservative calibration for the origin of the atelid crown group.

To account for uncertainty, we applied normal prior distributions with appropriate standard deviations of 0.5 was used for the calibration points. A total of four independent runs of 20 million generations were conducted. The convergence of the runs was evaluated in Tracer v.1.7 (Rambaut et al. 2018), where ESS values above 200 across all independent runs considered satisfactory and indicates that our divergence time estimates are robust and well-supported. The log and tree files were combined in LogCombiner v1.8.4 (Drummond et al. 2012). Finally, the tree file was summarized using TreeAnnotator v.1.8.4 (Drummond et al. 2012).

The choice of the four calibration points was based on their phylogenetic relevance, the availability of consistent fossil records, and their use in prior robust phylogenetic studies (Kiesling et al. 2015; Morales-Jiménez et al. 2015; Rosenberger et al. 2015). Using multiple independent calibration points assist reduce biases that can arise from relying on a single divergence event and allows for more accurate and stable time estimates across the phylogeny.

All the topologies generated by the different phylogenetic analyses were visualized and edited using FigTree 1.4.4 (Rambaut 2018).

## Results

### Database characterization and genetic distance

The concatenated data provided a database with a total of 16,093 bp (14,481 bp from nuclear DNA and 1,612 bp from mitochondrial DNA). A description of the molecular markers used is shown in Table S4, including the nucleotide substitution models chosen by Kakusan4, the number of variable and informative sites for parsimony, and the sizes of the markers. The mitochondrial markers showed higher numbers of variable and informative sites for parsimony than the nuclear markers.

The genetic distance obtained using the Kimura 2-Parameter (K2P) model, considering the concatenated data, had a mean of approximately 0.017 among the *Alouatta* species, ranging from 0.0028 (*A. discolor* vs. *A. belzebul*) to 0.0481 (*A. sara* vs. *A. pigra*) (Table 2). The genetic distance with mitochondrial DNA was around 7 times greater than the distance for nuclear DNA. Within *Alouatta*, the genetic distance for mitochondrial DNA had an average of 0.057, ranging from 0.0064 between *A. nigerrima* and *A. macconnelli* to 0.0816 between *A. palliata* and *A. sara* (Table S5). Nuclear DNA showed a lower average of 0.0075 between the species of the genus. The smallest distance was 0.0004 between *A. belzebul* and *A. discolor*, and the largest distance was 0.0134 between *A. pigra* and *A. discolor* (Table S6).

### Phylogenetic analysis

The results of the phylogenetic analyses (ML and BI) were congruent with each other in tree topology. In both Maximum Likelihood and Bayesian Inference analyses, seven of the nine species included in this study were recovered as well-supported monophyletic clades (*A. belzebul*, *A. guariba*, *A. caraya*, *A. nigerrima*, *A. macconnelli*, *A. sara*, and *A. palliata*) all showing maximum bootstrap (100) and posterior probability (1.0) values.

**Fig. 2:**
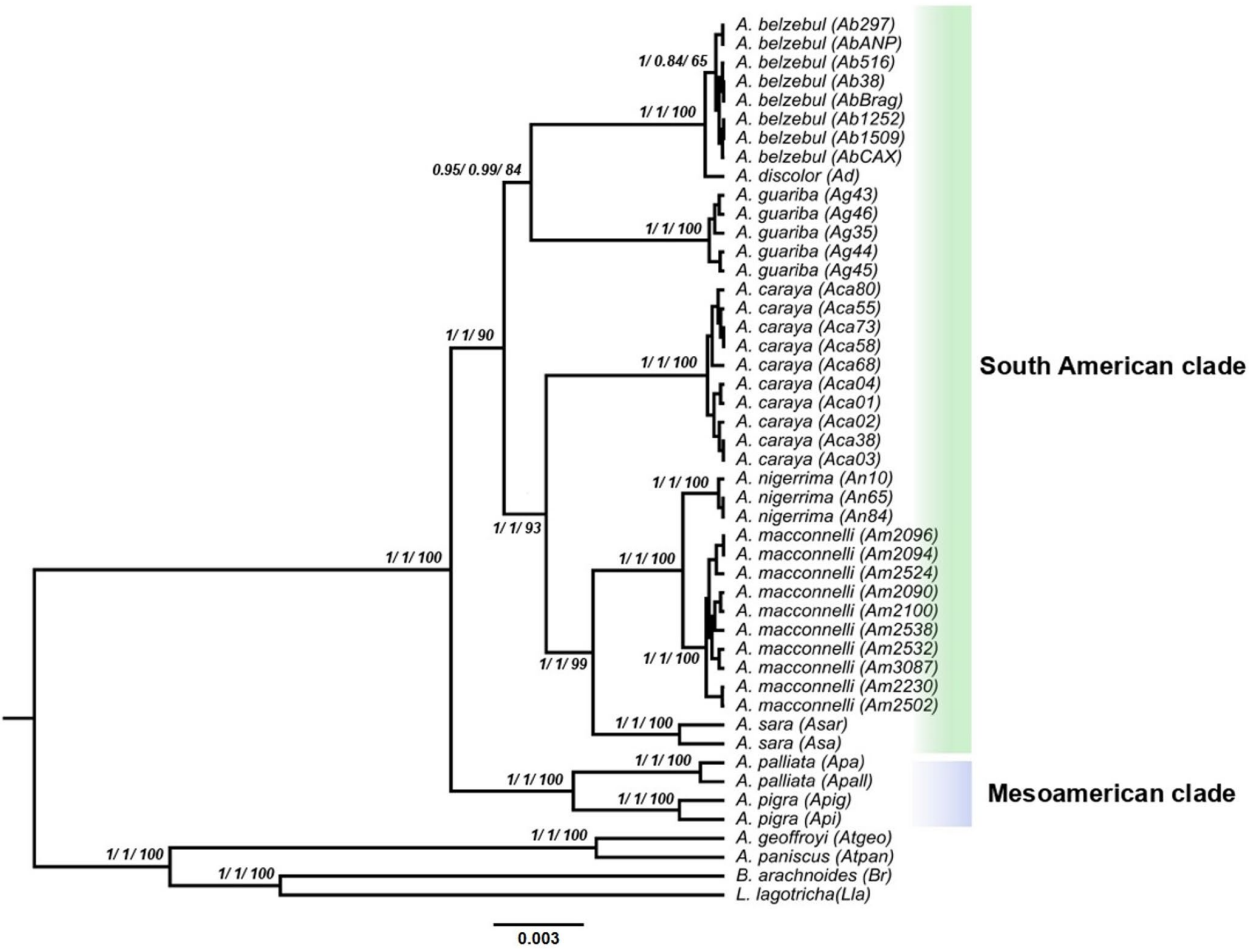
Phylogenetic tree estimated using ML and BI. For each node, there are the posterior probability values from MrBayes and BEAST, and on the right side, there are the bootstrap values from RAxML. Branch lengths are proportional to the number of nucleotide substitutions per site. The colored bars separate the *Alouatta* into two clades, South American (green) and Mesoamerican (blue)

The only species that was not recovered with a high support value was *A. belzebul*, a species closely related to *A. discolor*. The species *A. belzebul* was recovered with a bootstrap support of 65% and a PP of 0.84 (Fig. 2).

Our results provide high support for two clades within *Alouatta*: the South American and the Mesoamerican clades. Within the South American clade, there are two well-supported groups with reciprocal monophyly (Fig. 2). The first group is formed by the species of *A. guariba* and the *A. belzebul* complex (*A. belzebul* and *A. discolor*), supported with an 84% bootstrap and 0.99 PP. The second group was recovered with a support of 93% bootstrap and 1 PP, showing two monophyletic clades, one corresponding to the species *A. caraya*, and the other formed by the species complex *A. seniculus* (*A. macconnelli*, *A. nigerrima*, and *A. sara*) which, in this study, brings together the species *A. macconnelli* and *A. nigerrima* as sister species and the species *A. sara* as the most external to the clade (Fig. 2).

The Mesoamerican clade was composed of the species *A. palliata* and *A. pigra*. Each of these species and their relationship to each other were recovered with the maximum bootstrap and PP support. The phylogenetic trees inferred using MrBayes, BEAST, and RAxML were topologically congruent. The BEAST results provided maximum support for all *Alouatta* species (Fig. 2).

In addition, to assess the potential impact of marker type on phylogenetic inference, we also performed separate analyses using only mitochondrial DNA and only nuclear DNA markers. The resulting topologies were largely congruent with the concatenated dataset. Differences were observed in both BI and ML analyses based on mitochondrial DNA, in which *A. discolor* was recovered within the *A. belzebul* clade. In the nuclear dataset, however, *A. discolor* was recovered as the sister lineage to the *A. belzebul* clade, consistent with the topology observed in the concatenated analysis.

Additionally, a minor topological discrepancy was observed in the nuclear DNA Bayesian inference, where *A. sara* appeared as sister to *A. caraya*, contrasting with the Maximum Likelihood topology that grouped *A. sara* with *A. nigerrima* and *A. macconnelli* within the *A. seniculus* complex. Although the posterior probability supporting the *A. sara*-*A. caraya* relationship was moderate, this topology resembles that recovered by Doyle et al. (2021). In this regard, the topology obtained from the concatenated dataset appears to better reflect the taxonomic and biogeographic structure of Alouatta, presenting topologies similar to those found in more recent studies (Janiak et al. 2022; Povill et al. 2025; Schwantes et al. 2025) and reinforcing its interpretive robustness. These additional trees are available in the supplementary material. (Fig. S1).

### Divergence times

**Fig. 3:**
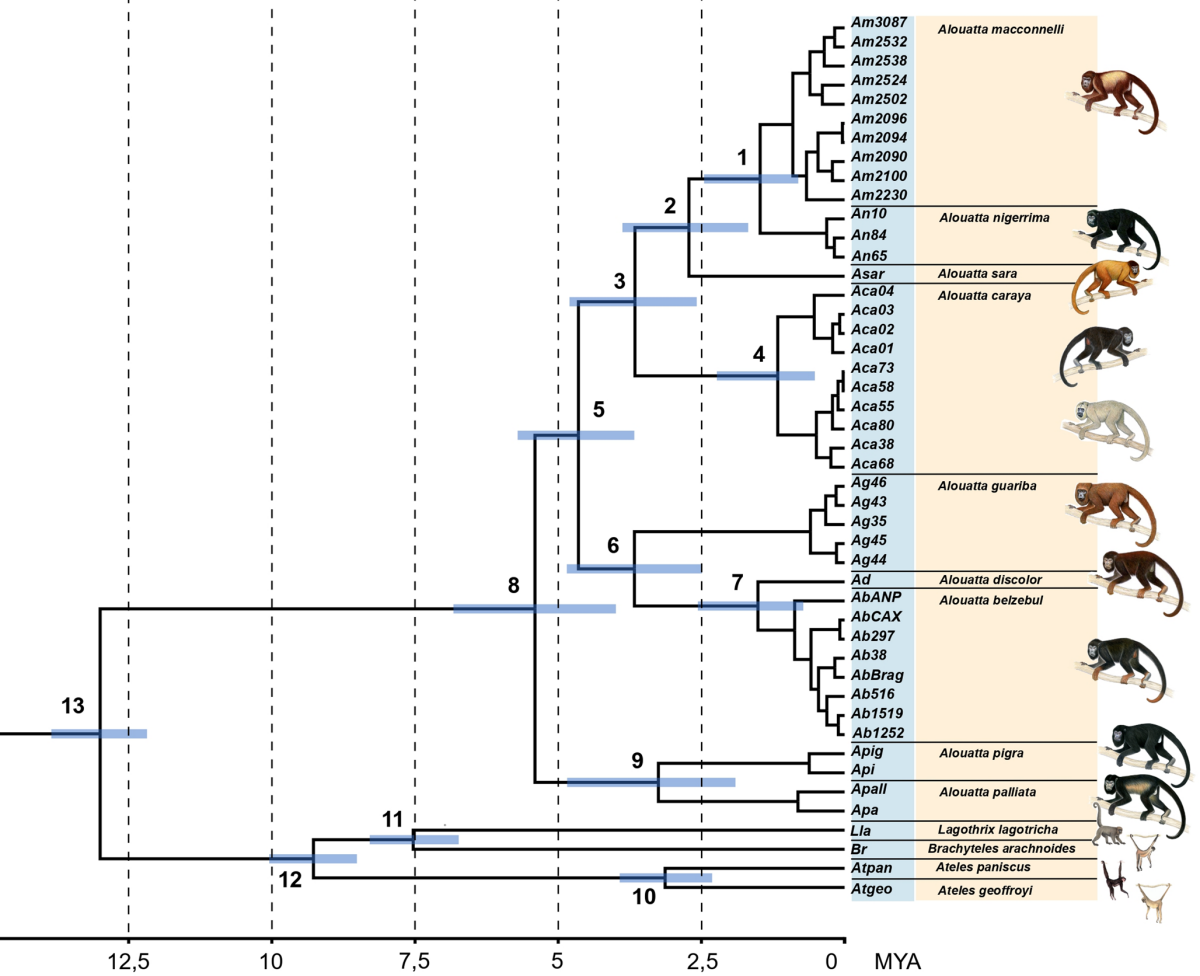
Phylogenetic relationships and divergence times estimated for Alouatta species done in the BEAST software, using concatenated mitochondrial and nuclear DNA sequences. The time of each node is referenced in Table 3

The divergence time analyses using a relaxed clock indicated an early divergence date for *Alouatta* at 5.60 Ma, with a 95% Highest Posterior Density (95% HPD) ranging from 3.99 to 6.83 Ma (Fig. 3). The initial separation of the genus resulted in a division into two clades, within the South American *Alouatta*, 4.47 Ma ago [HPD 95%, 3.68–5.34 Ma]. The two groups of South American *Alouatta* formed on this date diversified at close dates, the *A. belzebul* complex separated from *A. guariba* 3.82 Ma ago [95% HPD, 2.50–4.91 Ma], while *A. caraya* separated from the *A. seniculus* complex 3.62 Ma ago [95% HPD, 2.57–4.85 Ma]. Most speciation events occurred during the Pliocene. The most recent speciations were of *A. nigerrima/A. macconnelli* and *A. discolor/A. belzebul*, whose divergence would have occurred ~ 1.5 Ma ago (Table 3).

**Table 3:** Median node dates and 95% HPD intervals, in millions of years (Ma)

Node of the Fig. 3	Age (Ma)	95% HPD (Ma)
1	1.51	0.79–2.48
2	2.69	1.51–3.19
3	3.62	2.57–4.85
4	1.21	0.34–2.46
5	4.47	3.68–5.34
6	3.82	2.50–4.91
7	1.48	0.52–2.51
8	5.60	3.99–6.83
9	3.21	1.89–4.89
10	3.07	2.39–3.68
11	7.50	6.83–8.16
12	9.25	8.24–10.01
13	12.95	12.19–13.76

Estimated using concatenated mitochondrial and nuclear markers

## Discussion

### The origin of the Mesoamerican and South American clades

This study provides new insights into the phylogenetic relationship of howler monkeys, using nuclear and mitochondrial markers. By integrating these distinct markers, which differ in their modes of inheritance and evolutionary rates, the analysis assist to resolve systematic and phylogenetic uncertainties among howler monkey species, as it provides a more robust and detailed phylogenetic resolution than that obtained in other previous hypotheses, which were mainly based on mitochondrial markers (Janiak et al. 2022; Meireles et al. 1999; Povill et al. 2022, 2023, 2025; Villalobos et al. 2004), nuclear (Doyle et al. 2021), or both markers (Cortés-Ortiz et al. 2003; Schwantes et al. 2025).

In addition to confirming well-established clades, this study introduces new findings by integrating, multilocus data (nuclear and mitochondrial) for *A. discolor* and *A. nigerrima* from confirmed localities. Although the general topology recovered here is consistent with previous mitogenomic analyses (Janiak et al. 2022; Povill et al. 2025), our approach expands upon those studies by including nuclear data and a broader taxonomic sampling, allowing a more comprehensive and robust reconstruction of *Alouatta* relationships. Furthermore, the inclusion of these taxa, along with a broader multilocus dataset, contributes to a more resolved and robust phylogenetic framework, particularly for South American lineages.

The genus *Alouatta* is separated into two well-supported groups: the Mesoamerican and the South American clades. In general, our study and previous studies support the recovery of these distinct clades of howler monkeys (Cortés-Ortiz et al. 2003; Doyle et al. 2021; Kuderna et al. 2023; Schwantes et al. 2025; Villalobos et al. 2004).

The formation of these two main groups in the *Alouatta* phylogeny is congruent with the hypothesis that their diversification originated through Andean vicariance (Cortés-Ortiz et al. 2003). Dating analyses using the relaxed uncorrelated molecular clock resulted in an estimate of the time of diversification between the South American and Mesoamerican clades that coincides with the period of formation of the northern Andes (approximately 15–3 million years ago) (Hoorn et al. 2010; Lundberg et al. 1998).

This interpretation is also supported by different authors who dated the split between Mesoamerican and South American lineages at approximately ranging between 16 − 13 Mya, and associated it with ecological and fluvial rearrangements driven by the Andean uplift (Cortés-Ortiz et al. 2003; Doyle et al. 2021; Schwantes et al. 2025). Our study reinforces a more classical vicariant scenario, in which the physical rise of the Andes acted as a primary barrier, fragmenting an ancestral population and initiating the divergence between clades.

This interpretation contrasts with Schwantes et al. (2025), who propose that speciation in *Alouatta* predominantly followed a parapatric model, with ecological niche divergence acting as the main driver of diversification. While we acknowledge the potential role of ecological gradients, our findings support a more geographically structured model, in which physical barriers like the Andes and Amazon River played a decisive role in separating ancestral populations.

Additionally, the estimated date for the most recent common ancestor of the Mesoamerican clade is more recent than the time of speciation between the two main groups of the South American clade. In addition, our results indicate that the divergence of Mesoamerican *Ateles* and *Alouatta* lineages occurred prior to the complete closure of the Isthmus of Panama (~ 3 Ma), implying earlier biogeographic connections between South and Central America (Cortés-Ortiz et al. 2003; Morales-Jimenez et al. 2015).

These data reinforce the hypothesis that the evolutionary origin of the Mesoamerican howler monkeys can be explained by the invasion of a common ancestor from South America, via the land bridge connecting Central and South America, around 3.5 million years ago, a date that coincides with the divergence between *A. palliata* and *A. pigra* (Ellsworth and Hoelzer 2006; Hoorn et al. 2010; Smith 1970) (Fig. 3).

### The phylogenetic relationships and divergence of howler monkeys

The main speciation events that gave rise to *Alouatta* species or species groups occurred during the Pliocene epoch (2 to 5 million years ago), in agreement with the main biogeographic events in South America. Similar divergence dates are also seen in the genus *Ateles* and other new world primate genera (Janiak et al. 2022; Kiesling et al. 2015; Morales-Jimenez et al. 2015; Springer et al. 2012). In addition, some species pairs of howler monkeys have shown recent divergence dates, within the Pleistocene. These recent speciation events are observed, for example, in the sister species *A. macconnelli*/*A. nigerrima* and *A. belzebul*/*A. discolor*, which comprises the species with the most recent origin in *Alouatta*.

The divergence time estimates generated in this study help clarify the temporal disagreements reported in previous studies, which were based on limited datasets and single calibration points (e.g., Kiesling et al. 2015; Doyle et al. 2021). The improved agreement among our estimates results from combining multiple nuclear and mitochondrial markers with several independent calibrations, providing a more robust and coherent temporal framework for understanding the diversification of *Alouatta*.

It is important to mention here that this study has presented, for the first time, inferences based on nuclear and mitochondrial DNA sequences of the taxa *A. nigerrima* and *A. discolor* from known localities. These two species are closely related to *A. macconnelli* and *A. belzebul*, respectively. Some more recent studies considered *A. discolor* to be a valid species (Cortés-Ortiz et al. 2015a, b; Gregorin 2006; Kuderna et al. 2023), while others maintain *A. discolor* in the status as a subspecies of *A. belzebul* (e.g., Groves 2001; Rylands et al. 2000).

Although the genetic diversity observed for *A. belzebul* and *A. discolor* is relatively low, the separation of these species into distinct clades supports the hypothesis that they represent different evolutionary lineages. However, the incongruence between mitochondrial and nuclear datasets, with *A. discolor* clustering within the *A. belzebul* clade in the mtDNA tree but recovered as its sister lineage in the nuclear and concatenated analyses, suggests a more complex evolutionary history.

The differences between mitochondrial and nuclear phylogenetic trees are not unexpected, since these markers differ in mode of inheritance and evolutionary rate. Mitochondrial DNA reflects only the maternal lineage and may be more affected by introgression or lineage sorting, while nuclear loci represent biparental genomic history and typically recover species-level relationships more accurately when multiple loci are analyzed. Therefore, inconsistencies between mtDNA and nuclear topologies, such as those observed in our analyses, likely reflect these distinct evolutionary histories. Furthermore, such discrepancies may partly explain the inconsistencies among previous phylogenetic studies of *Alouatta*, many of which relied exclusively on mitochondrial or nuclear datasets.

In our study, this pattern may reflect mitochondrial introgression resulting from past hybridization between closely related populations, a phenomenon relatively common within the Platyrrhini clade (Cortés-Ortiz et al. 2015a, b, 2019; Martins-Junior et al. 2018; Vázquez-Domínguez et al. 2025), or incomplete lineage sorting due to a recent divergence (≈ 1.5 Ma) and the consequent retention of ancestral polymorphisms (Heads 2010; Perez et al. 2012, 2013). Both scenarios are consistent with the low genetic distances observed in previous studies (e.g., Schwantes et al. 2025), indicating that *A. discolor* likely originated from a recent radiation within the *A. belzebul* complex, followed by limited or asymmetric gene flow.

This same hypothesis can be applied to *A. nigerrima*, as a valid species closely related to *A. macconnelli*, with both diverging around 1.2 million years ago, a result that contradicts older taxonomic arrangements. (Cabrera 1958; Hershkovitz 1949; Hill 1962).

Regarding the Central American howler monkeys studied here, these are classified into two species, both of which are well-supported in our phylogeny. This result corroborates previous studies of *Alouatta* systematics, which support a sister species relationship between *A. palliata* and *A. pigra* (Cortés-Ortiz et al. 2003; Doyle et al. 2021; Ruiz-García et al. 2017). There are different hypotheses to explain the origin of these two sister species: the first involves a single dispersal to Central America and subsequent speciation of the two species from Mesoamerica; the second is a double invasion of Central America by ancestors from the Trans-Andean region of South America (Smith 1970). However, the true causes behind the separation of *A. palliata* and *A. pigra* are still unknown.

In the South American clade, the most recent diversification occurred around 5 Ma (Fig. 3). This event originated the common ancestor of *A. guariba* and *A. belzebul* complex and the ancestor of the sister group, *A. caraya* and *A. seniculus* complex. This date coincides with the formation of the transcontinental Amazon River, during the late Miocene and Pliocene (Albert et al. 2018). Thus, given the current geographic distribution of howler monkey species south and north of the Amazon River, the biogeographic scenario is in line with the rivers-as-barriers hypothesis (Wallace 1854) to explain this initial diversification of the South American *Alouatta* clade. This hypothesis was also used by Cortés-Ortiz et al. (2003) to explain this cladogenesis event among the South American *Alouatta*.

However, more recent interpretations, such as those presented by Schwantes et al. (2025), suggest that while rivers like the Amazon and Madeira may coincide temporally with diversification events, their role as strict geographic barriers may be overstated. Instead, they may have contributed to lineage divergence by promoting ecological differentiation and niche divergence across their margins. Despite this, our findings, particularly the deep genetic split and well-supported phylogenetic separation among taxa distributed on opposite sides of the Amazon, are consistent with a model in which the Amazon River played a historically significant role in structuring *Alouatta* diversity through geographic isolation.

Considering the group formed by *A. guariba* and the A. *belzebul* complex, this result is consistent with Cortés-Ortiz (2003) and Schwantes et al. (2025), who also recovered *A. guariba* as part of a well-supported clade that includes *A. belzebul* and other taxa associated with eastern Brazilian biomes. Both studies support the hypothesis that these lineages share a more recent common ancestor, likely shaped by habitat connections and subsequent fragmentation between the Amazon and Atlantic Forest during the Neogene (Cortés-Ortiz et al. 2003; Schwantes et al. 2025).

The *A. guariba* species has a distribution restricted to the Atlantic Forest, while the *A. belzebul* complex can occur in the eastern Amazon, the Caatinga and the Atlantic Forest of northeastern Brazil (Cortés-Ortiz et al. 2015a, b). The divergence between these lineages may have been driven by paleoclimatic events during the Late Miocene to Pliocene that caused forest retraction, savanna expansion, and the establishment of the South American dry diagonal. This environmental shift likely disrupted the historical forest corridor connecting the Amazon and Atlantic Forest, promoting geographic isolation and subsequent diversification of the group (Hoorn et al. 2010; Machado et al. 2021). Indeed, the current distribution of *A. belzebul* lineages along regions once covered by this forest corridor supports the hypothesis of a formerly continuous habitat that was fragmented by the development of the dry diagonal (Machado et al. 2021).

On the other hand, the second South American clade composed of *A. caraya* and the *A. seniculus* complex diverged 3.62 Ma ago. Among these species, *A. caraya* can be found in different parts of the South American continent, including the Cerrado, the Atlantic Rainforest, the Amazon, and areas of dry or humid savannas, and its distribution does not coincide with any clear geographical barrier (Bicca-Marques et al. 2008; Cortés-Ortiz et al. 2003). In addition, this species can occur sympatrically with other species, such as *A. guariba*, from the Atlantic Rainforest, where it can form hybrids (Bicca-Marques et al. 2008; Mourthé et al. 2019).

This species can also occur in sympatry with *A. sara*, but there are no records of these two species hybridizing (Büntge and Pyritz 2007). The phylogenetic position of *A. sara* also deserves attention. In our nuclear dataset, the Bayesian analysis recovered *A. sara* as sister to *A. caraya*, consistent with the topology proposed by Doyle et al. (2021), whereas the Maximum Likelihood analysis placed *A. sara* within the *A. seniculus* complex, together with *A. nigerrima* and *A. macconnelli*. This incongruence may reflect the limited number of informative sites supporting deep nodes within the complex, heterogeneous evolutionary rates among loci, or ancestral polymorphism retained across these closely related species.

Considering that the *A. seniculus* complex has undergone recent diversification and possible episodes of secondary contact, such topological variation among analyses is not unexpected. Additional multilocus data or genomic-scale analyses will be needed to fully resolve the position of *A. sara* within this complex.

A relevant fact that assist explain the formation and diversification of the South American clades of *Alouatta* is the expansion of forest areas that occurred during the late Miocene and early Pliocene, a period that coincides with the diversification of the groups of howler monkeys (Hoorn et al. 2010; Kay 2015; Schwantes et al. 2025). These connections between biomes that are currently separated, such as the Atlantic Rainforest and the Amazon, may have been important for some expansions of the range of *Alouatta*, where the clade formed by *A. belzebul* and *A. guariba* would have moved towards the Atlantic Rainforest (Batalha-Filho et al. 2013; Hoorn et al. 2010). The other clade consists of *A. caraya*,* A. nigerrima*,* A. macconnelli*, and *A. sara*, probably expanded in the opposite direction, towards savannas and flooded areas.

The *A. seniculus* complex, from the Amazon, is a group with high diversity in *Alouatta*, and due to difficulties in obtaining a wide taxonomic and geographical sample, many studies present this type of limitation in their results. The absence of *A. seniculus*, which is considered closer to *A. sara* rather than *A. macconnelli* (Cortés-Ortiz et al. 2003; Ruiz-Garcia et al. 2017), is a limitation of this study. However, our results confirm the taxonomic status of taxa recently elevated to species level, e.g., *A. sara* and *A. macconnelli*, previously treated as subspecies of *A. seniculus* (Hill 1962), and later considered valid species (Cortés-Ortiz et al. 2003; Minezawa et al. 1986).

Finally, our phylogenetic hypothesis, which supports the formation of two main groups within the South American *Alouatta* clade, is consistent with several molecular studies (Bonvicino et al. 2001; Cortés-Ortiz et al. 2003; Kuderna et al. 2023). However, other studies have recovered conflicting topologies (Doyle et al. 2021; Oliveira et al. 2002; Villalobos et al. 2004), likely due to limited taxonomic sampling and high levels of missing data in their datasets. Since in phylogenetic analyses, missing data, often due to partial sequences, incomplete gene sampling, or poor-quality alignments, can reduce statistical support and distort inferred relationships, especially when involving recent divergences or closely related taxa.

It is also important to note that part of the discrepancies among previous phylogenetic studies may reflect differences in sampling design rather than true topological conflict. Earlier analyses varied substantially in the number of species and loci included, as well as in the availability of georeferenced samples, which can influence both node support and inferred relationships. Therefore, some of the apparent inconsistencies in previous topologies likely result from reduced sampling or incomplete taxon representation, rather than genuine discordance among datasets. These issues likely contributed to the inconsistent topologies observed in previous studies compared to the more comprehensive multilocus approach adopted here.

## Conclusions

Our study corroborates the separation of the genus *Alouatta* into two groups: Mesoamerican and South American. In turn, the South American species of the genus *Alouatta* can be divided into two monophyletic groups, one that lists the *A. belzebul* complex and *A. guariba* as sister species and another group composed of *A. caraya* and the Amazonian species of the *A. seniculus* complex.

The main diversifications within the South American and Mesoamerican clades occurred during the Pliocene epoch. The relationship between the *A. belzebul* complex and *A. guariba* suggests a historical connection between the Amazon and the Atlantic Forest, while the *A. nigerrima* and *A. discolor* species can be considered distinct lineages with independent evolutionary histories, having their origins during the Pleistocene, and are among the species with the most recent origin and diversification within the genus.

In conclusion, while our findings broadly agree with earlier studies in recognizing major clades and some sister-species relationships, they diverge in important aspects such as the internal topology of South American lineages, the role of geographic versus ecological barriers, and the placement of Atlantic Forest taxa. By integrating broader molecular sampling, including nuclear and mitochondrial sequences from taxa such as *A. discolor* and *A. nigerrima* from confirmed localities, and clarifying debated relationships, our study contributes to a refined understanding of the evolutionary and biogeographic processes that shaped the diversification of *Alouatta*. However, it is important to mention that a better understanding of the evolutionary history of *Alouatta* will depend on further taxonomic, geographic, and genomic sampling.

## Supplementary Information

Below is the link to the electronic supplementary material.

Supplementary Material 1
