# Supplementary material for: Revisiting the relationships among howler monkeys through molecular phylogenetic analysis (Primates; Atelidae; Alouatta)
Source: Primates. 2026 Jan 20;67(2):225–37. doi: 10.1007/s10329-025-01233-0 (PMC12956992; doi:10.1007/s10329-025-01233-0)
Supplement: Supplementary file 1 — Supplementary Material 1 [file 10329_2025_1233_MOESM1_ESM.docx]

**Revisiting the relationships among howler monkeys through molecular phylogenetic analysis (Primates; Atelidae; *Alouatta*)**

*Primates*

Danillo Figueiredo da Silva^1^, Rodrigo Petry Corrêa de Sousa^1^, Adam Bessa-Silva^1^, Grazielle Fernanda Evangelista Gomes^2^, Marcelo Vallinoto^1^, Iracilda Sampaio^1^

^1^ Laboratório de Evolução, Instituto de Estudos Costeiros, Universidade Federal do Pará, Campus de Bragança, Bragança, PA, Brazil.

Corresponding author: [ira@ufpa.br](mailto:ira@ufpa.br)

**Table S1** Data on the species analyzed in this study obtained via GenBank, molecular markers, geographical origin, accession number and references.

| Markers | Species | Geographical origin | Accession number | References |
| --- | --- | --- | --- | --- |
| *m003* | *Alouatta palliata* | San Diego Zoo | GCA_004027835 | Johnson et al. (2018) |
| *m003* | *Alouatta palliata* | CRES | KC760246 | Kiesling et al. (2015) |
| *m003* | *Alouatta pigra* | México | MT903797 | Doyle et al. (2021) |
| *m003* | *Alouatta pigra* | México | MT903803 | Doyle et al. (2021) |
| *m003* | *Alouatta sara* | Unknown | MT903815 | Doyle et al. (2021) |
| *m003* | *Ateles geoffroyi* | San Diego Zoo | GCA_004024785 | Johnson et al. (2018) |
| *m003* | *Brachyteles arachnoides* | CPRJ | KC760250 | Kiesling et al. (2015) |
| *m254* | *Alouatta palliata* | San Diego Zoo | GCA_004027835 | Johnson et al. (2018) |
| *m254* | *Alouatta palliata* | Mexico | MT903877 | Doyle et al. (2021) |
| *m254* | *Alouatta pigra* | Mexico | MT903878 | Doyle et al. (2021) |
| *m254* | *Alouatta pigra* | Mexico | MT903879 | Doyle et al. (2021) |
| *m254* | *Ateles geoffroyi* | San Diego Zoo | GCA_004024785 | Johnson et al. (2018) |
| *m254* | *Brachyteles arachnoides* | CPRJ | KC761590 | Kiesling et al. (2015) |
| *m011* | *Alouatta palliata* | San Diego Zoo | GCA_004027835 | Johnson et al. (2018) |
| *m011* | *Alouatta palliata* | CRES | KC760370 | Kiesling et al. (2015) |
| *m011* | *Ateles geoffroyi* | San Diego Zoo | GCA_004024785 | Johnson et al. (2018) |
| *m011* | *Brachyteles arachnoides* | CPRJ | KC760374 | Kiesling et al. (2015) |
| *LRPPRC_169* | *Alouatta palliata* | San Diego Zoo | GCA_004027835 | Johnson et al. (2018) |
| *LRPPRC_169* | *Alouatta palliata* | San‐Diego, CA, US | HM761141 | Perelman et al. (2011) |
| *LRPPRC_169* | *Alouatta sara* | San‐Diego, CA, US | HM761142 | Perelman et al. (2011) |
| *LRPPRC_169* | *Ateles geoffroyi* | San Diego Zoo | GCA_004024785 | Johnson et al. (2018) |
| *m271* | *Alouatta palliata* | San Diego Zoo | GCA_004027835 | Johnson et al. (2018) |
| *m271* | *Alouatta palliata* | Mexico | MT903772 | Doyle et al. (2021) |
| *m271* | *Alouatta pigra* | Mexico | MT903774 | Doyle et al. (2021) |
| *m271* | *Alouatta pigra* | Mexico | MT903773 | Doyle et al. (2021) |
| *m271* | *Alouatta sara* | Unknown | MT903775 | Doyle et al. (2021) |
| *m271* | *Ateles geoffroyi* | San Diego Zoo | GCA_004024785 | Johnson et al. (2018) |
| *m271* | *Brachyteles arachnoides* | CPRJ | KC761772 | Kiesling et al. (2015) |
| *TTR* | *Alouatta palliata* | San Diego Zoo | GCA_004027835 | Johnson et al. (2018) |
| *TTR* | *Alouatta palliata* | San‐Diego, CA, US | HM757718 | Perelman et al. (2011) |
| *TTR* | *Alouatta sara* | San‐Diego, CA, US | HM757719 | Perelman et al. (2011) |
| *TTR* | *Ateles geoffroyi* | San Diego Zoo | GCA_004024785 | Johnson et al. (2018) |
| *TTR* | *Brachyteles arachnoides* | INCA | HM757704 | Perelman et al. (2011) |
| *GHR* | *Alouatta palliata* | San Diego Zoo | GCA_004027835 | Johnson et al. (2018) |
| *GHR* | *Alouatta palliata* | San‐Diego, CA, US | HM761468 | Perelman et al. (2011) |
| *GHR* | *Alouatta sara* | San‐Diego, CA, US | HM761464 | Perelman et al. (2011) |
| *GHR* | *Lagothrix lagotricha* | CCR | HM761491 | Perelman et al. (2011) |
| *GHR* | *Ateles paniscus* | FRZB | HM761490 | Perelman et al. (2011) |
| *GHR* | *Ateles geoffroyi* | San Diego Zoo | GCA_004024785 | Johnson et al. (2018) |
| *AXIN1* | *Alouatta palliata* | San Diego Zoo | GCA_004027835 | Johnson et al. (2018) |
| *AXIN1* | *Alouatta palliata* | San‐Diego, CA, US | HM764315 | Perelman et al. (2011) |
| *AXIN1* | *Alouatta sara* | San‐Diego, CA, US | HM764316 | Perelman et al. (2011) |
| *AXIN1* | *Lagothrix lagotricha* | CCR | HM764300 | Perelman et al. (2011) |
| *AXIN1* | *Ateles geoffroyi* | San Diego Zoo | GCA_004024785 | Johnson et al. (2018) |
| *AXIN1* | *Brachyteles arachnoides* | INCA | HM764298 | Perelman et al. (2011) |
| *m194* | *Alouatta palliata* | San Diego Zoo | GCA_004027835 | Johnson et al. (2018) |
| *m194* | *Alouatta palliata* | CRES | KC761127 | Kiesling et al. (2015) |
| *m194* | *Alouatta sara* | Unknown | MT903847 | Doyle et al. (2021) |
| *m194* | *Alouatta pigra* | Mexico | MT903846 | Doyle et al. (2021) |
| *m194* | *Ateles paniscus* | MGTC | KC761130 | Kiesling et al. (2015) |
| *m194* | *Ateles geoffroyi* | San Diego Zoo | GCA_004024785 | Johnson et al. (2018) |
| *m194* | *Brachyteles arachnoides* | CPRJ | KC761131 | Kiesling et al. (2015) |
| *m043_044* | *Alouatta palliata* | San Diego Zoo | GCA_004027835 | Johnson et al. (2018) |
| *m043_044* | *Alouatta palliata* | CRES | KC760664 | Kiesling et al. (2015) |
| *m043_044* | *Ateles paniscus* | MGTC | KC760667 | Kiesling et al. (2015) |
| *m043_044* | *Ateles geoffroyi* | San Diego Zoo | GCA_004024785 | Johnson et al. (2018) |
| *m043_044* | *Brachyteles arachnoides* | CPRJ | KC760668 | Kiesling et al. (2015) |
| *BRCA2* | *Alouatta palliata* | San Diego Zoo | GCA_004027835 | Johnson et al. (2018) |
| *BRCA2* | *Alouatta palliata* | San‐Diego, CA, US | HM763720 | Perelman et al. (2011) |
| *BRCA2* | *Alouatta sara* | San‐Diego, CA, US | HM763721 | Perelman et al. (2011) |
| *BRCA2* | *Ateles geoffroyi* | San Diego Zoo | GCA_004024785 | Johnson et al. (2018) |
| *BRCA2* | *Brachyteles arachnoides* | INCA | HM763704 | Perelman et al. (2011) |
| *mC17_01* | *Alouatta palliata* | San Diego Zoo | GCA_004027835 | Johnson et al. (2018) |
| *mC17_01* | *Alouatta palliata* | CRES | KC762030 | Kiesling et al. (2015) |
| *mC17_01* | *Lagothrix lagotricha* | BCNP | KC762033 | Kiesling et al. (2011) |
| *mC17_01* | *Ateles geoffroyi* | San Diego Zoo | GCA_004024785 | Johnson et al. (2018) |
| *m266* | *Alouatta palliata* | San Diego Zoo | GCA_004027835 | Johnson et al. (2018) |
| *m266* | *Alouatta palliata* | CRES | KC761711 | Kiesling et al. (2015) |
| *m266* | *Ateles geoffroyi* | San Diego Zoo | GCA_004024785 | Johnson et al. (2018) |
| *m266* | *Brachyteles arachnoides* | CPRJ | KC761715 | Kiesling et al. (2011) |
| *m263* | *Alouatta palliata* | San Diego Zoo | GCA_004027835 | Johnson et al. (2018) |
| *m263* | *Alouatta palliata* | CRES | KC761647 | Kiesling et al. (2015) |
| *m263* | *Ateles geoffroyi* | San Diego Zoo | GCA_004024785 | Johnson et al. (2018) |
| *m263* | *Brachyteles arachnoides* | CPRJ | KC761651 | Kiesling et al. (2015) |
| *m265* | *Alouatta palliata* | San Diego Zoo | GCA_004027835 | Johnson et al. (2018) |
| *m265* | *Alouatta palliata* | CRES | KC761678 | Kiesling et al. (2015) |
| *m265* | *Alouatta sara* | Unknown | MT903780 | Doyle et al. (2021) |
| *m265* | *Lagothrix lagotricha* | BCNP | KC761683 | Kiesling et al. (2015) |
| *m265* | *Ateles paniscus* | MGTC | KC761681 | Kiesling et al. (2015) |
| *m265* | *Ateles geoffroyi* | San Diego Zoo | GCA_004024785 | Johnson et al. (2018) |
| *m265* | *Brachyteles arachnoides* | CPRJ | KC761682 | Kiesling et al. (2015) |
| *m258* | *Alouatta palliata* | San Diego Zoo | GCA_004027835 | Johnson et al. (2018) |
| *m258* | *Alouatta palliata* | CRES | KC761616 | Kiesling et al. (2015) |
| *m258* | *Alouatta sara* | Unknown | MT903790 | Doyle et al. (2021) |
| *m258* | *Alouatta pigra* | Mexico | MT903787 | Doyle et al. (2021) |
| *m258* | *Alouatta pigra* | Mexico | MT903789 | Doyle et al. (2021) |
| *m258* | *Ateles paniscus* | MGCT | KC761619 | Kiesling et al. (2015) |
| *m258* | *Ateles geoffroyi* | San Diego Zoo | GCA_004024785 | Johnson et al. (2018) |
| *m258* | *Brachyteles arachnoides* | CPRJ | KC761620 | Kiesling et al. (2015) |
| *m220* | *Alouatta palliata* | San Diego Zoo | GCA_004027835 | Johnson et al. (2018) |
| *m220* | *Alouatta palliata* | CRES | KC761388 | Kiesling et al. (2015) |
| *m220* | *Ateles geoffroyi* | San Diego Zoo | GCA_004024785 | Johnson et al. (2018) |
| *m220* | *Brachyteles arachnoides* | CPRJ | KC761392 | Kiesling et al. (2015) |
| *mC18* | *Alouatta palliata* | San Diego Zoo | GCA_004027835 | Johnson et al. (2018) |
| *mC18* | *Alouatta palliata* | CRES | KC762089 | Kiesling et al. (2015) |
| *mC18* | *Ateles geoffroyi* | San Diego Zoo | GCA_004024785 | Johnson et al. (2018) |
| *DENND5A* | *Alouatta palliata* | San Diego Zoo | GCA_004027835 | Johnson et al. (2018) |
| *DENND5A* | *Alouatta palliata* | San‐Diego, CA, US | HM759271 | Perelman et al. (2011) |
| *DENND5A* | *Alouatta sara* | San‐Diego, CA, US | HM759272 | Perelman et al. (2011) |
| *DENND5A* | *Ateles geoffroyi* | San Diego Zoo | GCA_004024785 | Johnson et al. (2018) |
| *DENND5A* | *Brachyteles arachnoides* | INCA | HM759251 | Perelman et al. (2011) |
| *ADORA3* | *Alouatta palliata* | San Diego Zoo | GCA_004027835 | Johnson et al. (2018) |
| *ADORA3* | *Alouatta palliata* | San‐Diego, CA, US | HM765173 | Perelman et al. (2011) |
| *ADORA3* | *Alouatta sara* | San‐Diego, CA, US | HM765174 | Perelman et al. (2011) |
| *ADORA3* | *Brachyteles arachnoides* | INCA | HM765156 | Perelman et al. (2011) |
| *mC21_01* | *Alouatta palliata* | San Diego Zoo | GCA_004027835 | Johnson et al. (2018) |
| *mC21_01* | *Alouatta palliata* | CRES | KC762089 | Kiesling et al. (2015) |
| *mC21_01* | *Ateles geoffroyi* | San Diego Zoo | GCA_004024785 | Johnson et al. (2018) |
| *mC21_01* | *Brachyteles arachnoides* | CPRJ | KC762147 | Kiesling et al. (2015) |
| *mC13_04* | *Alouatta palliata* | San Diego Zoo | GCA_004027835 | Johnson et al. (2018) |
| *mC13_04* | *Alouatta palliata* | San‐Diego, CA, US | KC761936 | Perelman et al. (2011) |
| *mC13_04* | *Ateles geoffroyi* | San Diego Zoo | GCA_004024785 | Johnson et al. (2018) |
| *COX1* | *Alouatta palliata* | Costa Rica | OM328926 | Janiak et al. (2022) |
| *COX1* | *Alouatta sara* | Rondônia, Brazil | OM328917 | Janiak et al. (2022) |
| *CYTB* | *Alouatta sara* | Rondônia, Brazil | OM328917 | Janiak et al. (2022) |
| *CYTB* | *Alouatta pigra* | México | AY065885 | Cortés-Ortiz et al (2003) |
| *CYTB* | *Alouatta palliata* | Costa Rica | OM328926 | Janiak et al. (2022) |
| *CYTB* | *Ateles geoffroyi* | Costa Rica | OM328927 | Janiak et al. (2022) |
| *COX1* | *Ateles geoffroyi* | Costa Rica | OM328927 | Janiak et al. (2022) |

CRES - The Center for Reproduction of Endangered species at the San Diego zoo; INCA – Instituto Nacional do Câncer, Rio de Janeiro; MGCT - Morris Godman Tissue Collection; CPRJ - Centro de Primatologia do Rio de Janeiro; BCNP - Brasil Centro Nacional de Primatas; CCR - Coriell Cell Repositories, NJ, US; FRZB - Fundação RioZoo, Brasil.

**Table S2** GenBank codes of the sequences produced in this study.

| Markers | Voucher | Species | Accession number |
| --- | --- | --- | --- |
| ***m003*** | Ab297 | *Alouatta belzebul* | PQ800545 |
| ***m003*** | Ab516 | *Alouatta belzebul* | PQ800546 |
| ***m003*** | Ad | *Alouatta discolor* | PQ800547 |
| ***m003*** | Ag35 | *Alouatta guariba* | PQ800548 |
| ***m003*** | An65 | *Alouatta nigerrima* | PQ800549 |
| ***m003*** | Am2230 | *Alouatta macconnelli* | PQ800550 |
| ***m003*** | Aca01 | *Alouatta caraya* | PQ800551 |
| ***m003*** | Aca02 | *Alouatta caraya* | PQ800552 |
| ***m003*** | Lla | *Lagothrix lagotricha* | PQ800553 |
| ***m003*** | Atgeo | *Ateles geoffroyi* | PQ800554 |
| ***m011*** | Ab297 | *Alouatta belzebul* | PQ818530 |
| ***m011*** | Ad | *Alouatta discolor* | PQ818531 |
| ***m011*** | Ag35 | *Alouatta guariba* | PQ818532 |
| ***m011*** | An10 | *Alouatta nigerrima* | PQ818533 |
| ***m011*** | Am2230 | *Alouatta macconnelli* | PQ818534 |
| ***m011*** | Am2090 | *Alouatta macconnelli* | PQ818535 |
| ***m011*** | Aca01 | *Alouatta caraya* | PQ818536 |
| ***m011*** | Aca02 | *Alouatta caraya* | PQ818537 |
| ***m011*** | Aca80 | *Alouatta caraya* | PQ818538 |
| ***m011*** | Lla | *Lagothrix lagotricha* | PQ818539 |
| ***m011*** | Atpan | *Ateles paniscus* | PQ818540 |
| ***m194*** | Ab297 | *Alouatta belzebul* | PQ818541 |
| ***m194*** | Ab516 | *Alouatta belzebul* | PQ818542 |
| ***m194*** | Ag35 | *Alouatta guariba* | PQ818543 |
| ***m194*** | Ag44 | *Alouatta guariba* | PQ818544 |
| ***m194*** | An65 | *Alouatta nigerrima* | PQ818545 |
| ***m194*** | An84 | *Alouatta nigerrima* | PQ818546 |
| ***m194*** | Am2230 | *Alouatta macconnelli* | PQ818547 |
| ***m194*** | Am2502 | *Alouatta macconnelli* | PQ818548 |
| ***m194*** | Am2524 | *Alouatta macconnelli* | PQ818549 |
| ***m194*** | Am2100 | *Alouatta macconnelli* | PQ818550 |
| ***m194*** | Aca01 | *Alouatta caraya* | PQ818551 |
| ***m194*** | Aca03 | *Alouatta caraya* | PQ818552 |
| ***m194*** | Aca04 | *Alouatta caraya* | PQ818553 |
| ***m194*** | Aca38 | *Alouatta caraya* | PQ818554 |
| ***m194*** | Aca68 | *Alouatta caraya* | PQ818555 |
| ***m194*** | Lla | *Lagothrix lagotricha* | PQ818556 |
| ***m220*** | Ab297 | *Alouatta belzebul* | PQ818557 |
| ***m220*** | AbANP | *Alouatta belzebul* | PQ818558 |
| ***m220*** | Ag35 | *Alouatta guariba* | PQ818559 |
| ***m220*** | Ag45 | *Alouatta guariba* | PQ818560 |
| ***m220*** | An65 | *Alouatta nigerrima* | PQ818561 |
| ***m220*** | Am2230 | *Alouatta macconnelli* | PQ818562 |
| ***m220*** | Aca01 | *Alouatta caraya* | PQ818563 |
| ***m220*** | Aca03 | *Alouatta caraya* | PQ818564 |
| ***m220*** | Lla | *Lagothrix lagotricha* | PQ818565 |
| ***m220*** | Atpan | *Ateles paniscus* | PQ818566 |
| ***m254*** | Ab297 | *Alouatta belzebul* | PQ818567 |
| ***m254*** | Ad | *Alouatta discolor* | PQ818568 |
| ***m254*** | Ag35 | *Alouatta guariba* | PQ818569 |
| ***m254*** | An65 | *Alouatta nigerrima* | PQ818570 |
| ***m254*** | Am2230 | *Alouatta macconnelli* | PQ818571 |
| ***m254*** | Aca01 | *Alouatta caraya* | PQ818572 |
| ***m254*** | Aca38 | *Alouatta caraya* | PQ818573 |
| ***m254*** | Lla | *Lagothrix lagotricha* | PQ818574 |
| ***m254*** | Atpan | *Ateles paniscus* | PQ818575 |
| ***m258*** | Ab297 | *Alouatta belzebul* | PQ818576 |
| ***m258*** | Ab1509 | *Alouatta belzebul* | PQ818577 |
| ***m258*** | Ag35 | *Alouatta guariba* | PQ818578 |
| ***m258*** | An10 | *Alouatta nigerrima* | PQ818579 |
| ***m258*** | An65 | *Alouatta nigerrima* | PQ818580 |
| ***m258*** | Am2230 | *Alouatta macconnelli* | PQ818581 |
| ***m258*** | Am2532 | *Alouatta macconnelli* | PQ818582 |
| ***m258*** | Am2094 | *Alouatta macconnelli* | PQ818583 |
| ***m258*** | Aca01 | *Alouatta caraya* | PQ818584 |
| ***m263*** | Ab297 | *Alouatta belzebul* | PQ818585 |
| ***m263*** | Ab1509 | *Alouatta belzebul* | PQ818586 |
| ***m263*** | AbANP | *Alouatta belzebul* | PQ818587 |
| ***m263*** | AbBrag | *Alouatta belzebul* | PQ818588 |
| ***m263*** | Ad | *Alouatta discolor* | PQ818589 |
| ***m263*** | Ag35 | *Alouatta guariba* | PQ818590 |
| ***m263*** | An65 | *Alouatta nigerrima* | PQ818591 |
| ***m263*** | An84 | *Alouatta nigerrima* | PQ818592 |
| ***m263*** | Am2230 | *Alouatta macconnelli* | PQ818593 |
| ***m263*** | Am2502 | *Alouatta macconnelli* | PQ818594 |
| ***m263*** | Aca01 | *Alouatta caraya* | PQ818595 |
| ***m263*** | Aca38 | *Alouatta caraya* | PQ818596 |
| ***m263*** | Aca68 | *Alouatta caraya* | PQ818597 |
| ***m263*** | Lla | *Lagothrix lagotricha* | PQ818598 |
| ***m263*** | Atpan | *Ateles paniscus* | PQ818599 |
| ***m265*** | Ab297 | *Alouatta belzebul* | PQ818600 |
| ***m265*** | Ag35 | *Alouatta guariba* | PQ818601 |
| ***m265*** | Ag43 | *Alouatta guariba* | PQ818602 |
| ***m265*** | An65 | *Alouatta nigerrima* | PQ818603 |
| ***m265*** | An84 | *Alouatta nigerrima* | PQ818604 |
| ***m265*** | Am2230 | *Alouatta macconnelli* | PQ818605 |
| ***m265*** | Am3087 | *Alouatta macconnelli* | PQ818606 |
| ***m265*** | Aca01 | *Alouatta caraya* | PQ818607 |
| ***m266*** | Ab297 | *Alouatta belzebul* | PQ818608 |
| ***m266*** | Ab516 | *Alouatta belzebul* | PQ818609 |
| ***m266*** | Ab1509 | *Alouatta belzebul* | PQ818610 |
| ***m266*** | AbBrag | *Alouatta belzebul* | PQ818611 |
| ***m266*** | Ab38 | *Alouatta belzebul* | PQ818612 |
| ***m266*** | AbCAX | *Alouatta belzebul* | PQ818613 |
| ***m266*** | Ag35 | *Alouatta guariba* | PQ818614 |
| ***m266*** | Ag43 | *Alouatta guariba* | PQ818615 |
| ***m266*** | Ag44 | *Alouatta guariba* | PQ818616 |
| ***m266*** | Ag46 | *Alouatta guariba* | PQ818617 |
| ***m266*** | An65 | *Alouatta nigerrima* | PQ818618 |
| ***m266*** | An84 | *Alouatta nigerrima* | PQ818619 |
| ***m266*** | Am2532 | *Alouatta macconnelli* | PQ818620 |
| ***m266*** | Am2090 | *Alouatta macconnelli* | PQ818621 |
| ***m266*** | Am2094 | *Alouatta macconnelli* | PQ818622 |
| ***m266*** | Am3087 | *Alouatta macconnelli* | PQ818623 |
| ***m266*** | Aca01 | *Alouatta caraya* | PQ818624 |
| ***m266*** | Aca38 | *Alouatta caraya* | PQ818625 |
| ***m266*** | Aca68 | *Alouatta caraya* | PQ818626 |
| ***m266*** | Aca73 | *Alouatta caraya* | PQ818627 |
| ***m266*** | Lla | *Lagothrix lagotricha* | PQ818628 |
| ***m266*** | Atpan | *Ateles paniscus* | PQ818629 |
| ***m271*** | Ab297 | *Alouatta belzebul* | PQ818630 |
| ***m271*** | AbANP | *Alouatta belzebul* | PQ818631 |
| ***m271*** | AbBrag | *Alouatta belzebul* | PQ818632 |
| ***m271*** | Ab38 | *Alouatta belzebul* | PQ818633 |
| ***m271*** | AbCAX | *Alouatta belzebul* | PQ818634 |
| ***m271*** | Ad | *Alouatta discolor* | PQ818635 |
| ***m271*** | Ag35 | *Alouatta guariba* | PQ818636 |
| ***m271*** | Ag43 | *Alouatta guariba* | PQ818637 |
| ***m271*** | Ag46 | *Alouatta guariba* | PQ818638 |
| ***m271*** | An65 | *Alouatta nigerrima* | PQ818639 |
| ***m271*** | Am2230 | *Alouatta macconnelli* | PQ818640 |
| ***m271*** | Am2532 | *Alouatta macconnelli* | PQ818641 |
| ***m271*** | Am2538 | *Alouatta macconnelli* | PQ818642 |
| ***m271*** | Am2090 | *Alouatta macconnelli* | PQ818643 |
| ***m271*** | Aca01 | *Alouatta caraya* | PQ818644 |
| ***m271*** | Aca38 | *Alouatta caraya* | PQ818645 |
| ***m271*** | Aca55 | *Alouatta caraya* | PQ818646 |
| ***m271*** | Aca68 | *Alouatta caraya* | PQ818647 |
| ***m271*** | Aca73 | *Alouatta caraya* | PQ818648 |
| ***m271*** | Aca80 | *Alouatta caraya* | PQ818649 |
| ***m271*** | Lla | *Lagothrix lagotricha* | PQ818650 |
| ***m271*** | Atpan | *Ateles paniscus* | PQ818651 |
| ***m043_044*** | Ab297 | *Alouatta belzebul* | PQ818652 |
| ***m043_044*** | Ab1509 | *Alouatta belzebul* | PQ818653 |
| ***m043_044*** | AbCAX | *Alouatta belzebul* | PQ818654 |
| ***m043_044*** | Ag35 | *Alouatta guariba* | PQ818655 |
| ***m043_044*** | Ag43 | *Alouatta guariba* | PQ818656 |
| ***m043_044*** | Ag44 | *Alouatta guariba* | PQ818657 |
| ***m043_044*** | An10 | *Alouatta nigerrima* | PQ818658 |
| ***m043_044*** | An65 | *Alouatta nigerrima* | PQ818659 |
| ***m043_044*** | An84 | *Alouatta nigerrima* | PQ818660 |
| ***m043_044*** | Am2524 | *Alouatta macconnelli* | PQ818661 |
| ***m043_044*** | Am2096 | *Alouatta macconnelli* | PQ818662 |
| ***m043_044*** | Aca01 | *Alouatta caraya* | PQ818663 |
| ***m043_044*** | Aca04 | *Alouatta caraya* | PQ818664 |
| ***m043_044*** | Aca38 | *Alouatta caraya* | PQ818665 |
| ***m043_044*** | Aca55 | *Alouatta caraya* | PQ818666 |
| ***m043_044*** | Lla | *Lagothrix lagotricha* | PQ818667 |
| ***ADORA3*** | Ab297 | *Alouatta belzebul* | PQ818715 |
| ***ADORA3*** | Ab516 | *Alouatta belzebul* | PQ818716 |
| ***ADORA3*** | Ab1509 | *Alouatta belzebul* | PQ818717 |
| ***ADORA3*** | Ab1252 | *Alouatta belzebul* | PQ818718 |
| ***ADORA3*** | AbBrag | *Alouatta belzebul* | PQ818719 |
| ***ADORA3*** | AbCAX | *Alouatta belzebul* | PQ818720 |
| ***ADORA3*** | Ag35 | *Alouatta guariba* | PQ818721 |
| ***ADORA3*** | An10 | *Alouatta nigerrima* | PQ818722 |
| ***ADORA3*** | An65 | *Alouatta nigerrima* | PQ818723 |
| ***ADORA3*** | Am2230 | *Alouatta macconnelli* | PQ818724 |
| ***ADORA3*** | Am2524 | *Alouatta macconnelli* | PQ818725 |
| ***ADORA3*** | Aca01 | *Alouatta caraya* | PQ818726 |
| ***ADORA3*** | Lla | *Lagothrix lagotricha* | PQ818727 |
| ***ADORA3*** | Atpan | *Ateles paniscus* | PQ818728 |
| ***ADORA3*** | Atgeo | *Ateles geoffroyi* | PQ818729 |
| ***mC17_01*** | Ab297 | *Alouatta belzebul* | PQ818668 |
| ***mC17_01*** | Ab1509 | *Alouatta belzebul* | PQ818669 |
| ***mC17_01*** | AbBrag | *Alouatta belzebul* | PQ818670 |
| ***mC17_01*** | Ag35 | *Alouatta guariba* | PQ818671 |
| ***mC17_01*** | An65 | *Alouatta nigerrima* | PQ818672 |
| ***mC17_01*** | Am2230 | *Alouatta macconnelli* | PQ818673 |
| ***mC17_01*** | Am2090 | *Alouatta macconnelli* | PQ818674 |
| ***mC17_01*** | Am2096 | *Alouatta macconnelli* | PQ818675 |
| ***mC17_01*** | Am2100 | *Alouatta macconnelli* | PQ818676 |
| ***mC17_01*** | Am3087 | *Alouatta macconnelli* | PQ818677 |
| ***mC17_01*** | Aca01 | *Alouatta caraya* | PQ818678 |
| ***mC17_01*** | Aca04 | *Alouatta caraya* | PQ818679 |
| ***mC17_01*** | Atpan | *Ateles paniscus* | PQ818680 |
| ***mC18_03*** | Ab297 | *Alouatta belzebul* | PQ818681 |
| ***mC18_03*** | Ab1509 | *Alouatta belzebul* | PQ818682 |
| ***mC18_03*** | Ad | *Alouatta discolor* | PQ818683 |
| ***mC18_03*** | Ag35 | *Alouatta guariba* | PQ818684 |
| ***mC18_03*** | Ag43 | *Alouatta guariba* | PQ818685 |
| ***mC18_03*** | An10 | *Alouatta nigerrima* | PQ818686 |
| ***mC18_03*** | An65 | *Alouatta nigerrima* | PQ818687 |
| ***mC18_03*** | An84 | *Alouatta nigerrima* | PQ818688 |
| ***mC18_03*** | Am2230 | *Alouatta macconnelli* | PQ818689 |
| ***mC18_03*** | Aca01 | *Alouatta caraya* | PQ818690 |
| ***mC18_03*** | Lla | *Lagothrix lagotricha* | PQ818691 |
| ***mC18_03*** | Atpan | *Ateles paniscus* | PQ818692 |
| ***mC21_01*** | Ab297 | *Alouatta belzebul* | PQ818693 |
| ***mC21_01*** | Ag35 | *Alouatta guariba* | PQ818694 |
| ***mC21_01*** | Ag45 | *Alouatta guariba* | PQ818695 |
| ***mC21_01*** | An10 | *Alouatta nigerrima* | PQ818696 |
| ***mC21_01*** | An65 | *Alouatta nigerrima* | PQ818697 |
| ***mC21_01*** | An84 | *Alouatta nigerrima* | PQ818698 |
| ***mC21_01*** | Am2230 | *Alouatta macconnelli* | PQ818699 |
| ***mC21_01*** | Am2094 | *Alouatta macconnelli* | PQ818700 |
| ***mC21_01*** | Aca01 | *Alouatta caraya* | PQ818701 |
| ***mC21_01*** | Lla | *Lagothrix lagotricha* | PQ818702 |
| ***mC21_01*** | Atpan | *Ateles paniscus* | PQ818703 |
| ***mC13_04*** | Ab297 | *Alouatta belzebul* | PQ818704 |
| ***mC13_04*** | AbCAX | *Alouatta belzebul* | PQ818705 |
| ***mC13_04*** | Ag35 | *Alouatta guariba* | PQ818706 |
| ***mC13_04*** | An65 | *Alouatta nigerrima* | PQ818707 |
| ***mC13_04*** | An84 | *Alouatta nigerrima* | PQ818708 |
| ***mC13_04*** | Am2230 | *Alouatta macconnelli* | PQ818709 |
| ***mC13_04*** | Am2532 | *Alouatta macconnelli* | PQ818710 |
| ***mC13_04*** | Am2096 | *Alouatta macconnelli* | PQ818711 |
| ***mC13_04*** | Aca01 | *Alouatta caraya* | PQ818712 |
| ***mC13_04*** | Lla | *Lagothrix lagotricha* | PQ818713 |
| ***mC13_04*** | Atpan | *Ateles paniscus* | PQ818714 |
| ***AXIN1*** | Ab297 | *Alouatta belzebul* | PQ821932 |
| ***AXIN1*** | AbANP | *Alouatta belzebul* | PQ821933 |
| ***AXIN1*** | Ad | *Alouatta discolor* | PQ821934 |
| ***AXIN1*** | Ag35 | *Alouatta guariba* | PQ821935 |
| ***AXIN1*** | Ag43 | *Alouatta guariba* | PQ821936 |
| ***AXIN1*** | Ag45 | *Alouatta guariba* | PQ821937 |
| ***AXIN1*** | An65 | *Alouatta nigerrima* | PQ821938 |
| ***AXIN1*** | Am2230 | *Alouatta macconnelli* | PQ821939 |
| ***AXIN1*** | Am2502 | *Alouatta macconnelli* | PQ821940 |
| ***AXIN1*** | Aca01 | *Alouatta caraya* | PQ821941 |
| ***AXIN1*** | Aca04 | *Alouatta caraya* | PQ821942 |
| ***AXIN1*** | Atpan | *Ateles paniscus* | PQ821943 |
| ***BRCA2*** | Ab297 | *Alouatta belzebul* | PQ821944 |
| ***BRCA2*** | Ad | *Alouatta discolor* | PQ821945 |
| ***BRCA2*** | Ag35 | *Alouatta guariba* | PQ821946 |
| ***BRCA2*** | Ag43 | *Alouatta guariba* | PQ821947 |
| ***BRCA2*** | An65 | *Alouatta nigerrima* | PQ821948 |
| ***BRCA2*** | An84 | *Alouatta nigerrima* | PQ821949 |
| ***BRCA2*** | Am2230 | *Alouatta macconnelli* | PQ821950 |
| ***BRCA2*** | Am2502 | *Alouatta macconnelli* | PQ821951 |
| ***BRCA2*** | Am2090 | *Alouatta macconnelli* | PQ821952 |
| ***BRCA2*** | Aca01 | *Alouatta caraya* | PQ821953 |
| ***BRCA2*** | Aca38 | *Alouatta caraya* | PQ821954 |
| ***BRCA2*** | Aca73 | *Alouatta caraya* | PQ821955 |
| ***BRCA2*** | Lla | *Lagothrix lagotricha* | PQ821956 |
| ***BRCA2*** | Atpan | *Ateles paniscus* | PQ821957 |
| ***DENND5A*** | Ab297 | *Alouatta belzebul* | PQ821958 |
| ***DENND5A*** | Ab516 | *Alouatta belzebul* | PQ821959 |
| ***DENND5A*** | Ag35 | *Alouatta guariba* | PQ821960 |
| ***DENND5A*** | An65 | *Alouatta nigerrima* | PQ821961 |
| ***DENND5A*** | An84 | *Alouatta nigerrima* | PQ821962 |
| ***DENND5A*** | Am2230 | *Alouatta macconnelli* | PQ821963 |
| ***DENND5A*** | Am2524 | *Alouatta macconnelli* | PQ821964 |
| ***DENND5A*** | Am2532 | *Alouatta macconnelli* | PQ821965 |
| ***DENND5A*** | Am2090 | *Alouatta macconnelli* | PQ821966 |
| ***DENND5A*** | Aca01 | *Alouatta caraya* | PQ821967 |
| ***DENND5A*** | Aca04 | *Alouatta caraya* | PQ821968 |
| ***DENND5A*** | Aca38 | *Alouatta caraya* | PQ821969 |
| ***DENND5A*** | Lla | *Lagothrix lagotricha* | PQ821970 |
| ***DENND5A*** | Atpan | *Ateles paniscus* | PQ821971 |
| ***GHR*** | Ab297 | *Alouatta belzebul* | PQ821972 |
| ***GHR*** | Ag35 | *Alouatta guariba* | PQ821973 |
| ***GHR*** | Am2230 | *Alouatta macconnelli* | PQ821974 |
| ***GHR*** | Am2502 | *Alouatta macconnelli* | PQ821975 |
| ***GHR*** | Aca01 | *Alouatta caraya* | PQ821976 |
| ***LRPPRC_169*** | Ab297 | *Alouatta belzebul* | PQ821977 |
| ***LRPPRC_169*** | Ab516 | *Alouatta belzebul* | PQ821978 |
| ***LRPPRC_169*** | Ab1509 | *Alouatta belzebul* | PQ821979 |
| ***LRPPRC_169*** | Ab1252 | *Alouatta belzebul* | PQ821980 |
| ***LRPPRC_169*** | AbANP | *Alouatta belzebul* | PQ821981 |
| ***LRPPRC_169*** | Ad | *Alouatta discolor* | PQ821982 |
| ***LRPPRC_169*** | Ag35 | *Alouatta guariba* | PQ821983 |
| ***LRPPRC_169*** | An10 | *Alouatta nigerrima* | PQ821984 |
| ***LRPPRC_169*** | Am2230 | *Alouatta macconnelli* | PQ821985 |
| ***LRPPRC_169*** | Am2502 | *Alouatta macconnelli* | PQ821986 |
| ***LRPPRC_169*** | Am2524 | *Alouatta macconnelli* | PQ821987 |
| ***LRPPRC_169*** | Am2090 | *Alouatta macconnelli* | PQ821988 |
| ***LRPPRC_169*** | Am2094 | *Alouatta macconnelli* | PQ821989 |
| ***LRPPRC_169*** | Aca01 | *Alouatta caraya* | PQ821990 |
| ***LRPPRC_169*** | Aca03 | *Alouatta caraya* | PQ821991 |
| ***LRPPRC_169*** | Aca04 | *Alouatta caraya* | PQ821992 |
| ***LRPPRC_169*** | Aca38 | *Alouatta caraya* | PQ821993 |
| ***LRPPRC_169*** | Lla | *Lagothrix lagotricha* | PQ821994 |
| ***LRPPRC_169*** | Atpan | *Ateles paniscus* | PQ821995 |
| ***TTR*** | Ab297 | *Alouatta belzebul* | PQ821996 |
| ***TTR*** | Ab1509 | *Alouatta belzebul* | PQ821997 |
| ***TTR*** | Ab1252 | *Alouatta belzebul* | PQ821998 |
| ***TTR*** | AbBrag | *Alouatta belzebul* | PQ821999 |
| ***TTR*** | Ag35 | *Alouatta guariba* | PQ822000 |
| ***TTR*** | Ag43 | *Alouatta guariba* | PQ822001 |
| ***TTR*** | Ag45 | *Alouatta guariba* | PQ822002 |
| ***TTR*** | An10 | *Alouatta nigerrima* | PQ822003 |
| ***TTR*** | An65 | *Alouatta nigerrima* | PQ822004 |
| ***TTR*** | An84 | *Alouatta nigerrima* | PQ822005 |
| ***TTR*** | Am2230 | *Alouatta macconnelli* | PQ822006 |
| ***TTR*** | Am2502 | *Alouatta macconnelli* | PQ822007 |
| ***TTR*** | Am2524 | *Alouatta macconnelli* | PQ822008 |
| ***TTR*** | Am2532 | *Alouatta macconnelli* | PQ822009 |
| ***TTR*** | Aca01 | *Alouatta caraya* | PQ822010 |
| ***TTR*** | Lla | *Lagothrix lagotricha* | PQ822011 |
| ***TTR*** | Atpan | *Ateles paniscus* | PQ822012 |
| ***COX1*** | Ab297 | *Alouatta belzebul* | PQ837851 |
| ***COX1*** | Ab516 | *Alouatta belzebul* | PQ837852 |
| ***COX1*** | Ab1509 | *Alouatta belzebul* | PQ837853 |
| ***COX1*** | Ad | *Alouatta discolor* | PQ837854 |
| ***COX1*** | Ag35 | *Alouatta guariba* | PQ837855 |
| ***COX1*** | An65 | *Alouatta nigerrima* | PQ837856 |
| ***COX1*** | An84 | *Alouatta nigerrima* | PQ837857 |
| ***COX1*** | Am2230 | *Alouatta macconnelli* | PQ837858 |
| ***COX1*** | Am2502 | *Alouatta macconnelli* | PQ837859 |
| ***COX1*** | Am2090 | *Alouatta macconnelli* | PQ837860 |
| ***COX1*** | Aca01 | *Alouatta caraya* | PQ837861 |
| ***COX1*** | Aca03 | *Alouatta caraya* | PQ837862 |
| ***COX1*** | Aca38 | *Alouatta caraya* | PQ837863 |
| ***COX1*** | Lla | *Lagothrix lagotricha* | PQ837864 |
| ***COX1*** | Atpan | *Ateles paniscus* | PQ837865 |
| ***COX1*** | Br | *Brachyteles arachnoides* | PQ837866 |
| ***CYTB*** | Ab297 | *Alouatta belzebul* | PQ849579 |
| ***CYTB*** | Ab516 | *Alouatta belzebul* | PQ849580 |
| ***CYTB*** | Ab1509 | *Alouatta belzebul* | PQ849581 |
| ***CYTB*** | AbANP | *Alouatta belzebul* | PQ849582 |
| ***CYTB*** | Ab38 | *Alouatta belzebul* | PQ849583 |
| ***CYTB*** | Ad | *Alouatta discolor* | PQ849584 |
| ***CYTB*** | Ag35 | *Alouatta guariba* | PQ849585 |
| ***CYTB*** | An65 | *Alouatta nigerrima* | PQ849586 |
| ***CYTB*** | An84 | *Alouatta nigerrima* | PQ849587 |
| ***CYTB*** | Am2230 | *Alouatta macconnelli* | PQ849588 |
| ***CYTB*** | Am2502 | *Alouatta macconnelli* | PQ849589 |
| ***CYTB*** | Am2524 | *Alouatta macconnelli* | PQ849590 |
| ***CYTB*** | Am2090 | *Alouatta macconnelli* | PQ849591 |
| ***CYTB*** | Am3087 | *Alouatta macconnelli* | PQ849592 |
| ***CYTB*** | Aca01 | *Alouatta caraya* | PQ849593 |
| ***CYTB*** | Aca38 | *Alouatta caraya* | PQ849594 |
| ***CYTB*** | Aca55 | *Alouatta caraya* | PQ849595 |
| ***CYTB*** | Lla | *Lagothrix lagotricha* | PQ849596 |
| ***CYTB*** | Atpan | *Ateles paniscus* | PQ849597 |

**Table S3** Molecular markers, primer sequences, annealing temperatures and their respective references.

| Molecular markers | Foward Sequence | Reverse Sequence | Annealing | References |
| --- | --- | --- | --- | --- |
| *ADORA3* | ACCCCCATGTTTGGCTGGAA | GATAGGGTTCATCATGGAGTT | 54 °C | Horvath et al. (2008) |
| *AXIN1* | CTCTGCCTTCGCTGTACCGTCTAC | GACCCACCTTTCCTAATCCTTGTC | 54 °C | Horvath et al. (2008) |
| *BRCA2* | AGCTCTTTTGGGACAATTCTGAGGA | CTGTAATTTCTGCCTTTTGRCTAGG | 53 ºC | Perelman et al. (2011) |
| *DENND5A* | CCAGAGTTATCATGGCCAATC | GTACCAAGCAAGAAGCTGGG | 55 °C | Perelman et al. (2011) |
| *GHR* | CCAGTTCCAGTTCCAAAGAT | TGATTCTTCTGGTCAAGGCA | 59 ºC | Venta et al.  (1996) |
| *LRPPRC­169* | CTGGAGACAGCTTTTGTGTTATCC | ACCTTAGAGGCTGAAAGTGACTG | 57 °C | Horvath et al. (2008) |
| *TTR* | TGCCTCGCTGGACTGGTATT | GACAGCATCTAGAACTTTGACCAT | 58 °C | Flynn and Nedbal (1998) |
| *m003* | GCCTAAGATCTAATCAGCACATTG | CTGTATCTGCCTTTGTAAGAGGT | 59 °C | Kiesling et al. (2015) |
| *m011* | CAATGGATGGATGAGAG | GCAGAACACTCAGTGAGGTAC | 57 ºC | Kiesling et al. (2015) |
| *m043_044* | AAGGAACCACCACTAGGAG | GTGCAACATTTATCATGACTTC | 58 °C | Kiesling et al. (2015) |
| *m220* | CTCTGTGCTGGGAGACAAGG | TGTGGCAACAGAGTCAGGATTC | 58 º C | Kiesling et al. (2015) |
| *m254* | ACATATGACTCAGGCCAAATC | GCATTGCAGTAGCTAGCAC | 59 °C | Kiesling et al. (2015) |
| *m258* | CCAAGGCATAGTGTCTTAACA | AACCTGTCCCTGTATCTAAAAC | 59 º C | Kiesling et al. (2015) |
| *m263* | ACAAGTTAGCATCTGATTCATTTAC | CAACTTTCCTGGTAGCTTTGA | 56 º C | Kiesling et al. (2015) |
| *m265* | TCCATAGTACACCAAGGGACC | CCAATAGTATGCACTGTGAGCATG | 53 º C | Kiesling et al. (2015) |
| *m266* | CCACTAAGGAATTCTGATGCTT | GCATCACCCATGTATGATACATC | 58 º C | Kiesling et al. (2015) |
| *m194* | ATTCTATTCCCTGTGATGAWAGCAGA | TCTTTTCACTCAACATATGCCTGGA | 59 º C | Kiesling et al. (2015) |
| *m271* | CTTGAACAACACATATTTCCAACC | GAAAGATGGCATCTACTGGTGA | 55 º C | Kiesling et al. (2015) |
| *mC13_04* | CAGCCCAGAGTGCTTAGTTC | GAAGCAGATAGGCAGCCAC | 58 °C | Kiesling et al. (2015) |
| *mC17_01* | GTCCTGGATTTCCTATCACC | GGAGCTGTCTTCCTCTGTAA | 59º C | Kiesling et al. (2015) |
| *mC18_03* | GCATACATGCTTGCATCCAA | GCATAGTAGGGGTGCACAG | 58 °C | Kiesling et al. (2015) |
| *mC21_01* | GTCCAGTTGCTTATTAAGAGCAC | GATGACTAATTGATGTCAACAGGT | 58 °C | Kiesling et al. (2015) |
| *Cytochrome Oxidase I* | TTTGTTGGAGTARTATGTWACRAT | ATGTTCATAARYCGCTGAYTATT | 55 °C | This study |
| *Cytochrome Oxidase b* | ATCCAACATYTCCGCYTGATGA | GAAGGGGTATTCRACTGGTTGG | 55 °C | This study |

| **Molecular markers** | **Variable sites** | **Informative sites for parsimony** | **Size (bp)** | **Evolutionary models** |
| --- | --- | --- | --- | --- |
| ***m003*** | 28 (12) | 17 (10) | 480 | HKY |
| ***m011*** | 34 (12) | 28 (10) | 423 | HKY + G |
| ***m194*** | 27 (11) | 18 (9) | 426 | HKY + G |
| ***m220*** | 46 (15) | 32 (15) | 516 | K80 |
| ***m254*** | 37 (14) | 24 (13) | 436 | HKY |
| ***m258*** | 49 (24) | 33 (22) | 702 | HKY |
| ***m263*** | 33 (12) | 19 (8) | 485 | HKY + G |
| ***m265*** | 72 (24) | 51 (22) | 707 | HKY + G |
| ***m266*** | 59 (21) | 46 (19) | 757 | HKY + G |
| ***m271*** | 62 (28) | 42 (22) | 661 | HKY + G |
| ***m043_044*** | 78 (35) | 53 (32) | 801 | HKY + G |
| ***ADORA3*** | 21 (11) | 16 (9) | 387 | K80 + G |
| ***AXIN1*** | 30 (14) | 23 (12) | 892 | K80 + G |
| ***BRCA2*** | 57 (14) | 35 (11) | 969 | HKY |
| ***COXI*** | 177 (91) | 139 (82) | 712 | HKY + G |
| ***Cytb*** | 289 (162) | 204 (145) | 899 | HKY + G |
| ***mC17_01*** | 46 (20) | 33 (17) | 571 | K80 + G |
| ***mC18_03*** | 46 (26) | 29 (16) | 619 | HKY |
| ***mC21_01*** | 44 (9) | 31 (8) | 785 | HKY |
| ***mC13_04*** | 41 (12) | 31 (12) | 699 | HKY |
| ***DENND5A*** | 40 (9) | 21 (8) | 673 | K80 |
| ***GHR*** | 55 (14) | 30 (13) | 731 | HKY + G |
| ***LRPPRC_169*** | 41 (16) | 33 (15) | 837 | HKY + G |
| ***TTR*** | 57 (18) | 37 (14) | 925 | HKY + G |
| **Matched** | 1462 (616) | 10540 (544) | 16.093 | - |

**Table S4** Molecular markers, numbers of variable sites and informative sites for parsimony, fragment sizes (in bp), and their respective evolutionary models. Variable sites and informative sites for parsimony considering only the genus *Alouatta* are in the parentheses.

|  | 1 | 2 | 3 | 4 | 5 | 6 | 7 | 8 | 9 | 10 | 11 |
| --- | --- | --- | --- | --- | --- | --- | --- | --- | --- | --- | --- |
| 1 - *A. belzebul* |  |  |  |  |  |  |  |  |  |  |  |
| 2 - *A. discolor* | 0.0131 |  |  |  |  |  |  |  |  |  |  |
| 3 - *A. guariba* | 0.0436 | 0.0517 |  |  |  |  |  |  |  |  |  |
| 4 - *A. nigerrima* | 0.0548 | 0.0592 | 0.0505 |  |  |  |  |  |  |  |  |
| 5 - *A. macconnelli* | 0.0516 | 0.0564 | 0.0492 | 0.0064 |  |  |  |  |  |  |  |
| 6 - *A. caraya* | 0.0555 | 0.0576 | 0.0528 | 0.0491 | 0.0473 |  |  |  |  |  |  |
| 7 - *A. palliata* | 0.0751 | 0.0812 | 0.0710 | 0.0787 | 0.0770 | 0.0804 |  |  |  |  |  |
| 8 - *A. sara* | 0.049 | 0.0548 | 0.0562 | 0.0324 | 0.0302 | 0.0479 | 0.0816 |  |  |  |  |
| 9 - *A. pigra* | 0.0643 | 0.0677 | 0.0711 | 0.0745 | 0.0720 | 0.0706 | 0.0512 | 0.0693 |  |  |  |
| 10 - *L. lagotricha* | 0.153 | 0.160 | 0.161 | 0.164 | 0.161 | 0.162 | 0.169 | 0.173 | 0.171 |  |  |
| 11 - *A. paniscus* | 0.145 | 0.149 | 0.147 | 0.149 | 0.148 | 0.148 | 0.159 | 0.152 | 0.134 | 0.137 |  |
| 12 - *B. arachnoides* | 0.154 | 0.154 | 0.147 | 0.142 | 0.147 | 0.148 | 0.167 | 0.139 | 0.157 | 0.146 | 0.143 |

**Table S5** Mean genetic distance among *Alouatta* species and other genera of the family Atelidae (*Ateles paniscus*, *Brachyteles arachnoides,* and *Lagothrix lagotricha*). Based on mitochondrial markers, using the K2P model.

**Table S6.** Mean genetic distance among *Alouatta* species and other genera of the family Atelidae (*Ateles paniscus*, *Brachyteles arachnoides,* and *Lagothrix lagotricha*). Based on nuclear markers, using the K2P model.

|  | 1 | 2 | 3 | 4 | 5 | 6 | 7 | 8 | 9 | 10 | 11 |
| --- | --- | --- | --- | --- | --- | --- | --- | --- | --- | --- | --- |
| 1 - *A. belzebul* |  |  |  |  |  |  |  |  |  |  |  |
| 2 - *A. discolor* | 0.0004 |  |  |  |  |  |  |  |  |  |  |
| 3 - *A. guariba* | 0.0079 | 0.0071 |  |  |  |  |  |  |  |  |  |
| 4 - *A. nigerrima* | 0.0064 | 0.0067 | 0.0072 |  |  |  |  |  |  |  |  |
| 5 - *A. macconnelli* | 0.0073 | 0.0076 | 0.0081 | 0.0009 |  |  |  |  |  |  |  |
| 6 - *A. caraya* | 0.0077 | 0.0081 | 0.0089 | 0.0062 | 0.0073 |  |  |  |  |  |  |
| 7 - *A. palliata* | 0.0113 | 0.0101 | 0.0117 | 0.0103 | 0.0109 | 0.0117 |  |  |  |  |  |
| 8 - *A. sara* | 0.0043 | 0.0041 | 0.0067 | 0.0044 | 0.0033 | 0.0037 | 0.0011 |  |  |  |  |
| 9 - *A. pigra* | 0.0095 | 0.0134 | 0.0094 | 0.0108 | 0.0106 | 0.0117 | 0.0067 | 0.007 |  |  |  |
| 10 - *L. lagotricha* | 0.0319 | 0.0290 | 0.0337 | 0.0307 | 0.0327 | 0.0332 | 0.0345 | 0.0322 | 0.0313 |  |  |
| 11 - *A. paniscus* | 0.0329 | 0.0330 | 0.0337 | 0.0320 | 0.0329 | 0.0329 | 0.0350 | 0.0270 | 0.0309 | 0.0238 |  |
| 12 - *Brachyteles* | 0.0333 | 0.0323 | 0.0340 | 0.0324 | 0.0329 | 0.0334 | 0.0354 | 0.0284 | 0.0346 | 0.0188 | 0.0214 |


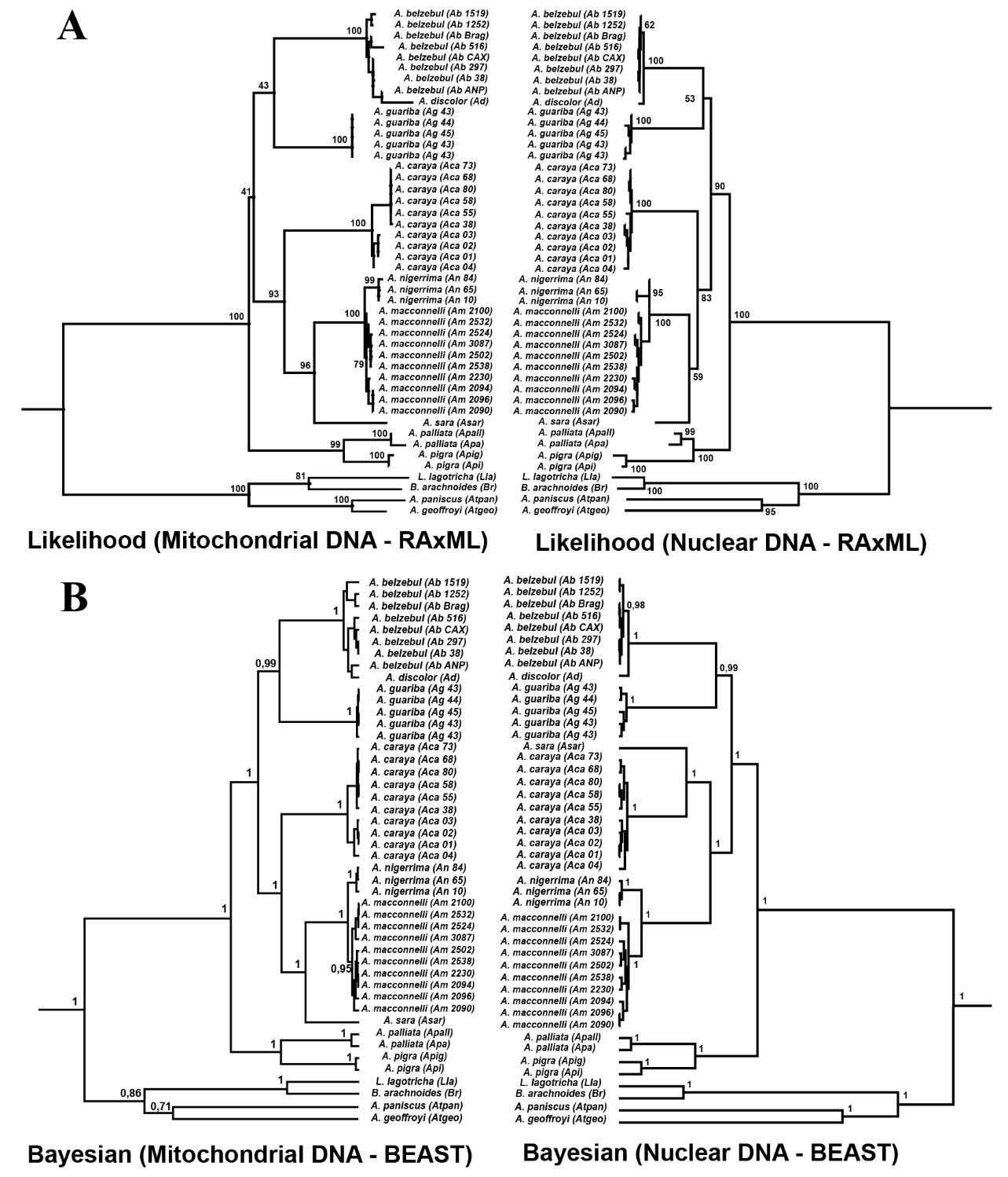


**Figure S1.** Phylogenetic trees representing the evolutionary relationships among species of the genus *Alouatta*, based on mitochondrial and nuclear DNA data. (A) Topologies inferred using the Maximum Likelihood method (RAxML), with statistical support indicated by bootstrap values. (B) Topologies inferred using Bayesian Inference (BEAST), with posterior probability values shown on the branches. On the left side of each panel are the analyses based on mitochondrial DNA; on the right side, analyses based on nuclear DNA.
